# Statistical perspective on functional and causal neural connectomics: The Time-Aware PC algorithm

**DOI:** 10.1371/journal.pcbi.1010653

**Published:** 2022-11-14

**Authors:** Rahul Biswas, Eli Shlizerman

**Affiliations:** 1 Department of Statistics, University of Washington, Seattle, Washington, United States of America; 2 Department of Applied Mathematics and Department of Electrical & Computer Engineering, University of Washington, Seattle, Washington, United States of America; Georgia State University, UNITED STATES

## Abstract

The representation of the flow of information between neurons in the brain based on their activity is termed the *causal functional connectome*. Such representation incorporates the dynamic nature of neuronal activity and causal interactions between them. In contrast to connectome, the causal functional connectome is not directly observed and needs to be inferred from neural time series. A popular statistical framework for inferring causal connectivity from observations is the *directed probabilistic graphical modeling*. Its common formulation is not suitable for neural time series since it was developed for variables with independent and identically distributed static samples. In this work, we propose to model and estimate the causal functional connectivity from neural time series using a novel approach that adapts directed probabilistic graphical modeling to the time series scenario. In particular, we develop the *Time-Aware PC* (TPC) algorithm for estimating the causal functional connectivity, which adapts the PC algorithm—a state-of-the-art method for statistical causal inference. We show that the model outcome of TPC has the properties of reflecting causality of neural interactions such as being non-parametric, exhibits the *directed Markov* property in a time-series setting, and is predictive of the consequence of counterfactual interventions on the time series. We demonstrate the utility of the methodology to obtain the causal functional connectome for several datasets including simulations, benchmark datasets, and recent multi-array electro-physiological recordings from the mouse visual cortex.

## Author summary

The *functional connectome* maps the connections that neurons utilize for information flow in the brain. Such a flow is dynamic and could be expressed as a “cause-and-effect” interaction between neurons. Adding these aspects to the functional connectome corresponds to specifying the direction of connections, i.e., mapping the *causal functional connectome*. Such a mapping is expected to lead to a more fundamental understanding of brain function and dysfunction. Among statistical frameworks to infer causal relations from observations, directed probabilistic graphical modeling is popular, however, its common formulation is not suitable for neurons since it was developed for variables with independent and identically distributed static samples, whereas neurons are dynamic objects generating time series of activity. In this work, we develop a novel approach for modeling and estimating causal functional connectome by adapting directed probabilistic graphical modeling to the time series scenario. We show its properties of reflecting causal interactions over time and demonstrate its utility in obtaining the causal functional connectome for different datasets including simulations, benchmark datasets, and recent multi-array electro-physiological recordings from the mouse visual cortex. With the article, an open-source Python package is made available. The package provides a full implementation of the methods along with examples.

This is a *PLOS Computational Biology* Methods paper.

## 1 Introduction

*Functional Connectome* (FC) refers to the network of interactions between units of the brain, such as individual neurons or brain regions, with respect to their activity over time [1]. The aim of finding the FC is to provide insight into how neurons interact to form brain function. FC can be represented by a graph whose nodes represent neurons and edges indicate a relationship between the activity of connected neurons. The edges can either represent undirected stochastic associations between activity of neurons or directed causal relationships between activity of neurons. While association between neural activity describes whether neuron *A* and neuron *B* are active in a correlated manner, the ultimate goal of functional connectomics is to answer causal queries, such as whether the activity in neuron *A* causes neuron *B* to be active (*A* → *B*), or is it the other way around (*B* → *A*)? Else, does a neuron *C* intermediate the correlation between *A* and *B* (*A* ← *C* → *B*) [2–4]?

When the interactions are causal, the network is termed as *causal functional connectome* (CFC). The CFC maps how neural activity flows within neural circuits, and provides the possibility for inference of neural pathways essential for brain functioning and behavior, such as sensory-motor-behavioral pathways [5]. Several approaches aim to infer CFC, such as Granger Causality (GC), Dynamic Causal Modeling (DCM), and Directed Probabilistic Graphical Models (DPGM), each having their applicability and challenges, as surveyed in [6]. GC obtains the directed functional connectivity from observed neural activity in a way that tells whether a neuron’s *past* is predictive of another neuron’s future, however it is unclear whether the prediction implies causation. In contrast, DCM compares *specific* mechanistic biological models based on data evidence, in which, model parameters represent causal influences between hidden neural states [7]. On the other hand, DPGM is a generic procedure to obtain causal relationships between nodes of a network from observations, using the *directed Markov* property. DPGM is non-parametric in the sense of capturing arbitrary functional relationships among the nodes and is predictive of the consequence of counterfactual interventions to the network [8, 9]. Such properties make DPGM a popular approach for causal modeling in various disciplines such as genomics and econometrics [10–21].

The utility of DPGM in obtaining CFC from neural data has been investigated in [6]. DPGM, applicable to independent and identically distributed (i.i.d.) observations, can model causal relations between whole time series of different neurons in sense of average over time or at a specific time. Thereby, standard DPGM does not explicitly model the inter-temporal causal relations between neural activity in the time series. For inference of the DPGM, the Peter and Clark (PC) algorithm is one of the widely used causal inference algorithms that assumes i.i.d. sampling of the nodes of the network and absence of latent confounders [22, 23]. However, in neural time series scenario, causal relations are between neural activity at different times. Since the nodes correspond to a time series with temporal dependency, the assumption in common DPGM of independent sampling of nodes is not suitable. Moreover, DPGM typically generate a Directed Acyclic Graph (DAG), while neural activity is often comprised of feedback loops over time [24–27]. Though adaptations aim to include cycles in the DPGM they have a more complicated output [28]. Addressing these limitations will improve the utility of DPGM for finding CFC in the neural time series scenario and is the focus of this work.

In this work, we develop a novel approach for modeling and estimating causal functional connectivity by adapting directed probabilistic graphical models to the time series scenario. We introduce the *Time-Aware PC* (TPC) algorithm. It uses the PC algorithm as a starting point and adapts it to the neural time series setup by following processes such as time-delay, bootstrapping and pruning. These ensure that the inferred CFC is robust and well suited to the setup. The proposed CFC graphical model incorporates feedback loops in functional connectivity and is non-parametric, yet we show that the CFC graphical model accurately represents the causal relationships in the unknown dynamical process of neural activity. Furthermore, the proposed CFC graphical model is predictive of the consequence of counterfactual interventions, such as the alteration in the CFC due to ablation or external control of certain neurons. We apply the proposed methodology on neural signals simulated from different paradigms and CFC motifs and demonstrate the utility of TPC in recovering the generating motifs. We also apply the TPC on public benchmark datasets and compare the performance in recovery of the ground truth CFC with other approaches. We further demonstrate the use of TPC to obtain the CFC among sampled neurons in mice brain from electrophysiological neural signals.

The following is a list of acronyms used in this paper: Functional Connectivity (FC), Causal Functional Connectivity (CFC), Granger Causality (GC), Dynamic Causal Model (DCM), Directed Probabilistic Graphical Model (DPGM), Directed Markov Property (DMP), Functional Magnetic Resonance Imaging (fMRI), Probabilistic Graphical Model (PGM), i.i.d. (Independent and Identically Distributed), Markov Property (MP), Directed Acyclic Graph (DAG), Peter and Clark (PC), Greedy Equivalence Search (GES), Greedy Interventional Equivalence Search (GIES), Continuous Time Recurrent Neural Network (CTRNN), True Positive (TP), False Positive (FP), True Negative (TN), False Negative (FN), True Positive Rate (TPR), False Positive Rate (FPR).

## 2 Causal functional connectivity for time series

In this section, we propose a novel methodology for modeling and estimation of the CFC for time series. This methodology is generic and applicable to various time series including neural recordings. The CFC is represented by a graph with nodes as neurons, each corresponds to a time series of neural activity, and edges indicating causal connectivity. In the following, we show we can explicitly model causal relations within and between time series in a DPGM framework and use it to define the CFC.

### 2.1 Unrolled graphical modeling of time series

**Fig 1:**
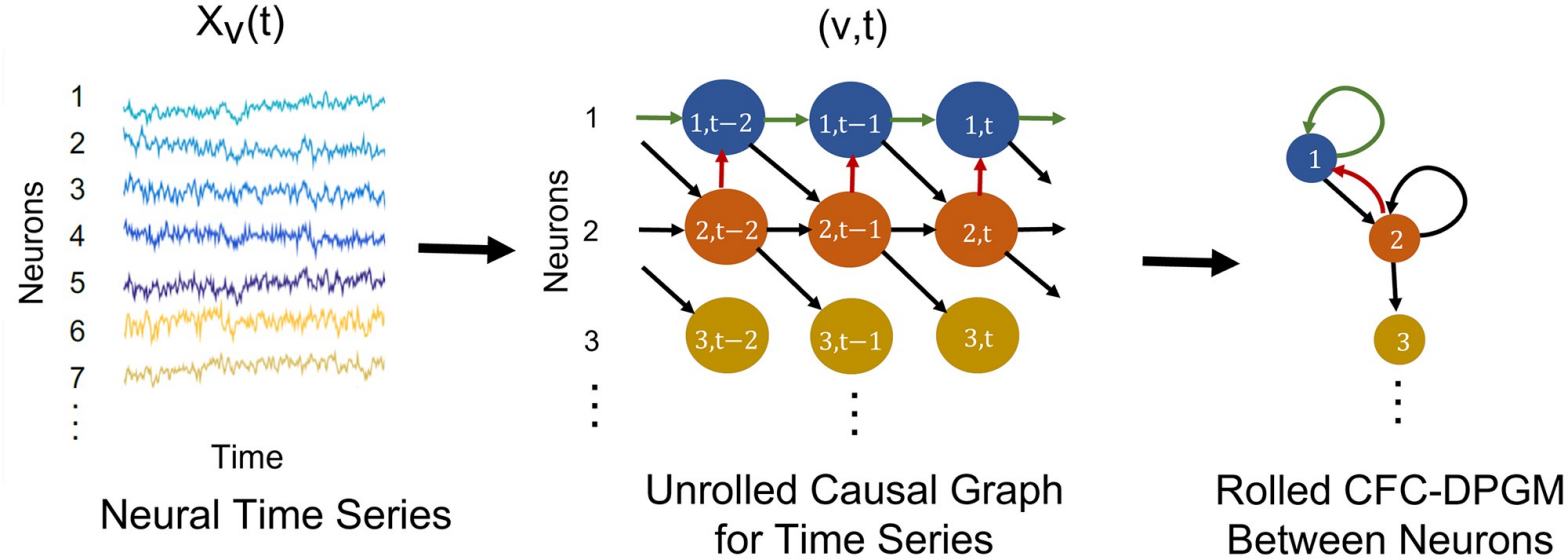
Causal modeling of neural time series. Schematic depiction of causal modeling of the neural time series (left) by the *Unrolled Causal Graph* (middle), and then rolling back its edges (colored) to define the *Rolled CFC-DPGM* (right).

We aim to incorporate the causal influence of the activity of neuron *u* at time *t*_1_ upon the activity of neuron *v* at time *t*_2_ in our proposed model. To do that, we *unroll* the time series *X*_*v*_(*t*), *v* ∈ *V*, *t* ∈ {0, …, *T*} into nodes (*v*, *t*) for neuron *v* and time *t* where the node (*v*, *t*) corresponds to the variable *X*_*v*_(*t*). We use directed edges between the nodes (*v*, *t*) to represent causal relations between *X*_*v*_(*t*). For example, the edge (*u*, *t*_1_)→(*v*, *t*_2_) represents the causal influence of the activity of neuron *u* at time *t*_1_, *X*_*u*_(*t*_1_) upon the activity of neuron *v* at time *t*_2_, *X*_*v*_(*t*_2_). Let ***V*** = {(*v*, *t*), *v* ∈ *V*, *t* ∈ {0, …, *T*}} be the set of nodes in the unrolled time series, ***E*** be the set of directed edges between the nodes and ***G*** = (***V***, ***E***) be the *Unrolled Graph* for the time series (See Fig 1-middle). We assume that ***G*** is a DAG in which there are no cycles in the edges in *E*.

Causal relationships between neurons are either forward in time: (1) from neurons *u* at time *t*_1_ to neuron *v* at time *t*_2_ for *t*_1_ < *t*_2_, represented by (*u*, *t*_1_) → (*v*, *t*_2_) for *t*_1_ < *t*_2_ in ***G***; or, (2) contemporaneous: when causal influences occur more rapidly than the sampling interval of the time series, represented in ***G*** by (*u*, *t*) → (*v*, *t*) at time *t* [29, 30]. Causal relations cannot direct backward in time, that is, ***G*** will not contain (*u*, *t*_1_) → (*v*, *t*_2_) for *t*_1_ > *t*_2_. Furthermore, for the contemporaneous causal influences, we do not allow the activity of *u* at time *t* to have causal influence on itself at time *t*, that is (*u*, *t*) → (*u*, *t*) is not allowed, while (*u*, *t*) → (*v*, *t*) is allowed. These considerations imply the absence of cycles in ***G***, thereby justifying the assumption for ***G*** to be a DAG to model the causal interactions in the unrolled time series.

In practice, the true causal interactions between *X*_*v*_(*t*) are unknown. Yet, when ***X*** = {*X*_*v*_(*t*) : (*v*, *t*) ∈ ***V***} satisfies the Directed Markov Property (DMP) with respect to DAG ***G*** (See Section 5.1 for details about the DMP), then it implies that ***G*** captures the causal functional interactions among *X*_*v*_(*t*), as we show in the functional equivalence of DMP in Section 5.1. We refer to such a DAG ***G*** as the *Unrolled Causal Graph* for the time series *X*_*v*_(*t*), *v* ∈ *V*, *t* ∈ {0, …, *T*}.

### 2.2 Rolled CFC-DPGM

Typically for signals, effective CFC representation refers to relations between neurons rather than relations between different signals’ times. Thereby, we propose to roll back the unrolled causal DAG ***G*** to define CFC between neurons (See Fig 1-right). The rolled graph is based on the principle that the existence of a causal relationship from neuron *u* at time *t*_1_ to neuron *v* at time *t*_2_, (*u*, *t*_1_) → (*v*, *t*_2_) ∈ ***G*** for *t*_1_ < *t*_2_, would imply that *u* is connected to *v* in the rolled CFC. In practice, the causal interactions weaken as the time-gap *t*_2_ − *t*_1_ grows. Thereby, we consider a maximum time-delay of interaction, *τ*, so that (*u*, *t*_1_) and (*v*, *t*_2_) would not share a significant influence between them if the time gap *t*_2_ − *t*_1_ > *τ* for all neurons *u*, *v*. Such a consideration of the maximum time-delay aids in making statistical inference from the time series data. Thus, if (*u*, *t*_1_) → (*v*, *t*_2_) in ***G*** for some *t*_1_ ≤ *t*_2_ ≤ *t*_1_ + *τ*, then the CFC graph between neurons should include *u* → *v*. We consolidate these concepts to define causal functional connectivity between neurons based on DPGM, in the following.

**Definition 1** (Rolled CFC-DPGM). Let ***X*** satisfy the DMP with respect to DAG ***G*** = (***V***, ***E***). The *Rolled CFC-DPGM* for neurons in *V* with maximum time-delay of interaction *τ*, is defined as the directed graph *F*_*τ*_ having edge *u* → *v* if (*u*, *t*_1_) → (*v*, *t*_2_) ∈ ***E*** for some *t*_1_ ≤ *t*_2_ ≤ *t*_1_ + *τ*, *t*_1_, *t*_2_ ∈ {0, 1, …, *T*}. *u*, *v* ∈ *V* could be either the same neuron or distinct neurons.

We show an example in Fig 1, where for neurons *V* = {1, 2, 3}, their unrolled DAG ***G*** is represented by Fig 1-middle. When the neural data ***X*** satisfies the directed Markov Property with respect to ***G***, the CFC graph with maximum time delay of interaction *τ* = 1 is given by Fig 1-right. Note that the same CFC graph would be obtained by taking any value of *τ* ≥ 1.

#### Contemporaneous and Feedback Interactions

We highlight that the Rolled CFC-DPGM in Definition 1 incorporates contemporaneous interactions, which can arise if the causal influences occur more rapidly than the sampling interval of the time series or the aggregation interval for aggregated time series. Such a scenario can arise for example in spiking neural datasets where peri-stimulus time histograms aggregate the spike trains over time intervals [31, 32], and in Functional Magnetic Resonance Imaging (fMRI) datasets where low sampling rates is typical [33]. However, if the time span in sampling and aggregation is expected to be less than the time scale of causal interactions, then one can impose *t*_1_ < *t*_2_ to exclude contemporaneous interactions. Additionally, the Rolled CFC-DPGM accomodates self-loops in neural interactions [34, 35], by checking whether (*u*, *t*_1_) → (*u*, *t*_2_) ∈ ***E*** for some 0 ≤ *t*_1_ < *t*_2_ ≤ *t*_1_ + *τ* in determining whether *u* → *u* ∈ *F*_*τ*_. Longer feedback loops are also incorporated. For example, the existence of *u* → *v* → *u* ∈ *F*_*τ*_ is determined by checking whether (*u*, *t*_1_) → (*v*, *t*_2_) and (*v*, *t*_2_) → (*u*, *t*_3_) ∈ ***E*** for some 0 ≤ *t*_1_ ≤ *t*_2_ ≤ *t*_3_ ≤ *t*_1_ + *τ*. By virtue of the technique of unrolling and rolling back, the Rolled CFC-DPGM captures causality while including cycles, since the Unrolled Graph is still a DAG and thereby meets the requirement for satisfying DMP.

### 2.3 Estimation from data: Time-Aware PC (TPC) algorithm

In this section we outline the steps to estimate the Rolled CFC-DPGM from time series *X*_*v*_(*t*), *v* ∈ *V*, *t* ∈ {0, 1, …, *T*}, dim*V* = *N*, which constitute the Time-Aware PC (TPC) Algorithm. Let us denote the recordings from the *N* neurons at time *t* by $X_{t}=\left(X_{v_{1}}\left(t\right),\ldots ,X_{v_{N}}\left(t\right)\right)$, where $\text{dim}\;X_{t}=N$, for *t* ∈ {0, 1, …, *T*}. The output of TPC is a Rolled CFC that contains edges found significant in the duration of recording. The TPC algorithm first aims to estimate the unrolled DAG with set of nodes {(*v*, *t*) : *v* ∈ *V*, *t* ∈ {0, 1, …, *τ*}}, for a fixed *τ* ≥ 1. For the estimation, TPC constructs samples $\chi_{k}=\left(X_{2(\tau +1)k},\ldots ,X_{\tau +2(\tau +1)k}\right)$, where $\text{dim}\;\chi_{k}=N\left(\tau +1\right)$, $k=0,1,\ldots ,K:=\lfloor \frac{T-\tau }{2(\tau +1)}\rfloor$. Note that $\chi_{k}$ is a concatenation of the vectors $X_{2(\tau +1)k},\ldots ,X_{\tau +2(\tau +1)k}$, each of dimension *N*, into a resulting vector of dimension *N*(*τ* + 1). We consider that the entry of $\chi_{k}$ corresponding to node *v* and time *t* + 2(*τ* + 1)*k*, i.e. *X*_*v*_(*t* + 2(*τ* + 1)*k*), for *k* = 0, …, *K* are samples for node (*v*, *t*), *v* ∈ *V*, *t* ∈ {0, 1, …, *τ*}. In other words, samples for a node (*v*, *t*) consists of *X*_*v*_(*t* + 2(*τ* + 1)*k*) for *k* = 0, 1, …, *K*, which are time advanced instances of neuron *v* starting at time *t* with time advances of multiples of 2(*τ* + 1). Such a time-advance of multiples of 2(*τ* + 1) ensures a substantial time-gap of 2(*τ* + 1) units between samples, that reduces interdependence between the samples, considering the maximum time-delay of interaction *τ* (See Section 5.2). This operation increases the dimension of the considered time-series at each time by a multiple of *τ* + 1 such that for original recordings of ***X***_*t*_ of dimension *N*, $\chi_{k}$ would have a dimension of *N*(*τ* + 1).

**Algorithm 1:** TPC

**Input :** Recordings of activity of neurons in *V* = {*v*_1_, …, *v*_N_} over time: $X_{t}=\left(X_{v_{1}}\left(t\right),\ldots ,X_{v_{N}}\left(t\right)\right)$, *t* ∈ {0, …, *T*}; maximum delay of interaction between neurons *τ*; significance level *α*; For bootstrapping: window width *L*; *m* iterations; bootstrap stability cutoff *γ*;

**Output**: CFC estimate, denoted *F*_*τ*_.

**begin**

 Step 1. Time-advanced samples. Let $\chi_{k}=\left(X_{2(\tau +1)k},\ldots ,X_{\tau +2(\tau +1)k}\right)$, where $\text{dim}\;\chi_{k}=N\left(\tau +1\right)$, $k=0,1,\ldots ,K:=\lfloor \frac{T-\tau }{2(\tau +1)}\rfloor$. Construct ${\left\{\chi_{k}\right\}}_{k=0}^{K}$.

 Step 2. Bootstrap. Select a random integer *n*_*b*_ ∈ {0, …, *K* − *L*} and choose the window of length *L* given by {*n*_*b*_ + 1, …, *n*_*b*_ + *L*} ⊆ {0, …, *K*}, to obtain ${\left\{\chi_{k}\right\}}_{k=n_{b}+1}^{n_{b}+L}$.

 Step 3. PC. Use the PC algorithm based on samples ${\left\{\chi_{k}\right\}}_{k=n_{b}+1}^{n_{b}+L}$ to estimate the unrolled DAG with nodes (*v*, *t*), *v* ∈ *V*, *t* ∈ {0, …, *τ*}. Denote the output as $G_{\tau }^{b}$.

 Step 4. Orient. Reverse the edge directions of edges $\left(v,t_{1}\right)\to \left(u,t_{2}\right)\in G_{\tau }^{b}$, when *t*_2_ < *t*_1_, *u* ∈ *V*, *v* ∈ *V* and update $G_{\tau }^{b}$.

 Step 5. Rolled CFC-DPGM.

  (a) Convert $G_{\tau }^{b}$ to rolled CFC-DPGM $F_{\tau }^{b}$ with max. delay of interaction *τ* using Def. 1.

  (b) Find the connectivity weights $w_{\tau }^{b}\left(u,v\right)$ for connections $u\to v\in F_{\tau }^{b}$ by Def. 5.3.

 Step 6. Robust edges.

  (a) Repeat Steps 2–5 to obtain *m* iterates of the rolled CFC-DPGM: $F_{\tau }^{b}$, and connectivity weights $w_{\tau }^{b}\left(u,v\right)$, *b* = 1, …, *m*.

  (b) Output a single CFC, *F*_*τ*_, with only those edges whose relative frequency of occurrence among $F_{\tau }^{b}$ is above *γ*.

  (c) Output single Connectivity Weights, *w*_*τ*_(*u*, *v*), for connections *u* → *v* ∈ *F*_*τ*_, as the average of $\left\{w_{\tau }^{b}\left(u,v\right):u\to v\in F_{\tau },u\to v\in F_{\tau }^{b},i\in 1,\ldots ,m\right\}$ when the set is non-empty and 0 otherwise.

 Step 7. Pruning. Remove from *F*_*τ*_ those edges *u* → *v* ∈ *F*_*τ*_ with |*w*_*τ*_(*u*, *v*)| < *w*_0_, where, $w_{0}=\frac{1}{10}\text{max}\{|w_{\tau }\left(u,v\right)\left|:u\to v\in F_{\tau }\right\}$.

**end**

In Step 2, TPC selects *m* windows of length *L* starting from *n*_*b*_ chosen at random, i.e. {*n*_*b*_ + 1, *n*_*b*_ + 2, …, *n*_*b*_ + *L*} ⊂ {0, 1, …, *N*}, to obtain ${\left\{\chi_{k}\right\}}_{k=n_{b}+1}^{n_{b}+L}$, *b* = 1, …, *m*. The process is called *bootstrap* since in the next step, the PC algorithm is applied on each subsample ${\left\{\chi_{k}\right\}}_{k=n_{b}+1}^{n_{b}+L}$ to output an estimate of the unrolled DAG with nodes (*v*, *t*), *v* ∈ *V*, *t* ∈ {0, 1, …, *τ*}, thus yielding a set of such estimates.

In Step 3, on each subsample ${\left\{\chi_{k}\right\}}_{k=n_{b}+1}^{n_{b}+L}$, the PC algorithm estimates the unrolled DAG with nodes (*v*, *t*), *v* ∈ *V*, *t* ∈ {0, 1, …, *τ*} as a completed partially directed acyclic graph (CPDAG), defined as the graph union of DAGs that satisfy DMP with respect to the subsample, and denoted by $G_{\tau }^{b}$ [36].

In Step 4, TPC *corrects* the edges in $G_{\tau }^{b}$ which direct from future to past time by reversing them, to be consistent with the temporal direction of causal interactions in the time series.

In Step 5, the re-oriented Unrolled graph $G_{\tau }^{b}$ is transformed to give the Rolled CFC-DPGM denoted $F_{\tau }^{b}$. At this step, weights for edges in $F_{\tau }^{b}$ are also obtained using interventional connectivity weights (5.3), that quantifies the causal effect of intervention on each neuron to its connected neurons.

In Step 6, a single CFC consensus *F*_*τ*_ is obtained by keeping only those edges which have relative frequency of occurrence among $F_{\tau }^{b},\;b=1,\ldots ,m$ to be greater than the cut-off *γ*. A single connectivity weight consensus for an edge in *F*_*τ*_ is achieved by averaging over the weights for the same edge, whenever present, over the *m* iterates. The resampling procedure promotes detection of stable edges [37].

**Fig 2:**
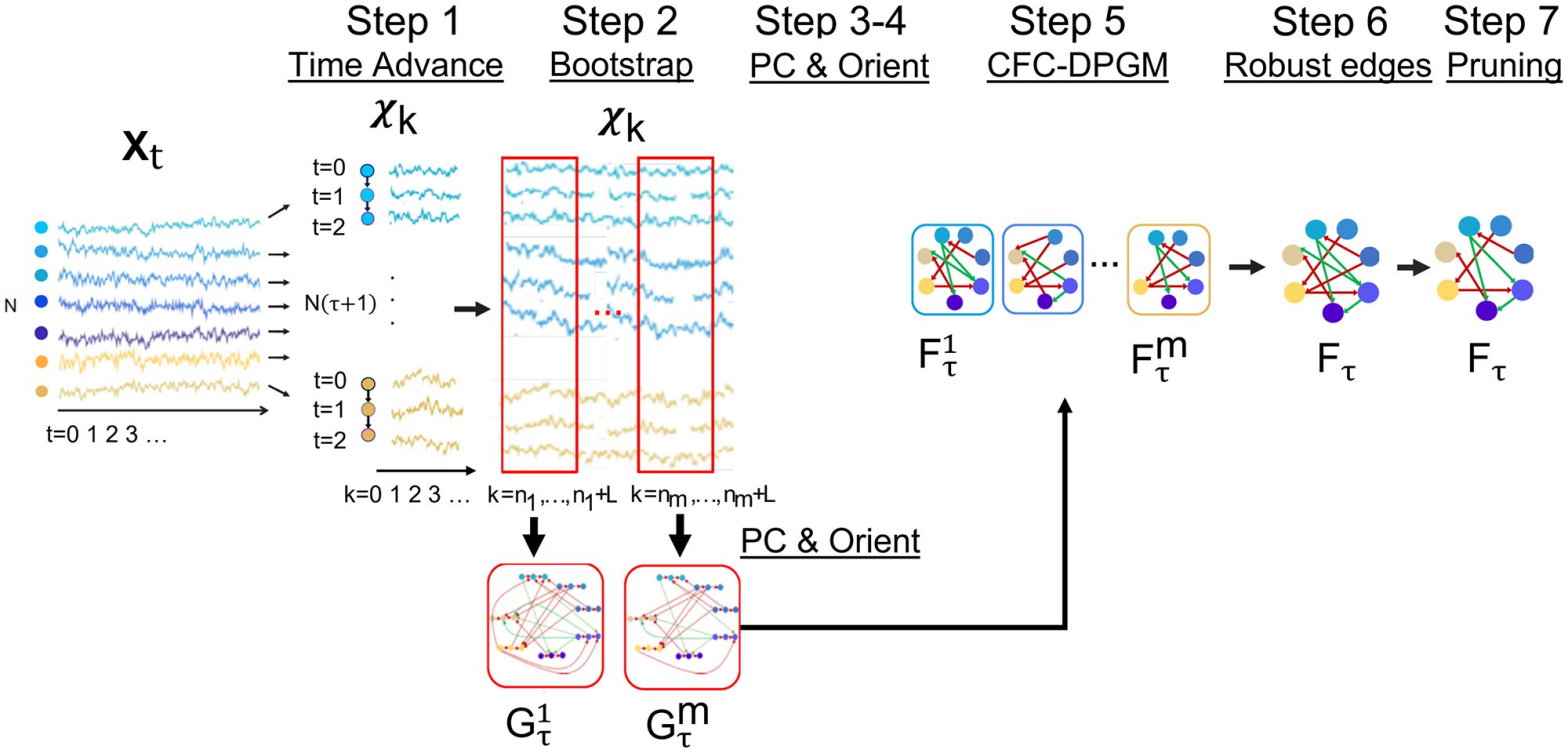
The TPC algorithm. Illustration of Steps 1–7 in the TPC Algorithm: Time Advance, Bootstrap, PC, Orient, Rolled CFC-DPGM, Robust edges and Pruning.

Finally, in Step 7, *F*_*τ*_ is pruned to further reduce spurious edges, by removing the edges which have exceedingly low connectivity weights, determined by those edges in *F*_*τ*_ whose weights are less than a tenth in magnitude compared to the maximum magnitude for edge weights in *F*_*τ*_. The TPC algorithm is outlined in Alg. 1 and Fig 2.

## 3 Results

### 3.1 Comparison study of TPC with other approaches to causal functional connectivity

We compare the performance of TPC with different existing CFC inference approaches to recover relationships in ground truth dynamical equations by generating synthetic data from three simulation paradigms. In particular, we estimate their CFC using GC, DPGM and TPC. The simulation paradigms correspond to specific model assumptions to assess the impact of model assumptions on the performance of the approaches (See Section C in S1 Appendix).

We measured the algorithms’ performance using CFC inference for 25 different simulations and summarized the results using three metrics: (1) Combined Score (CS), (2) True Positive Rate (TPR), (3) 1—False Positive Rate (IFPR). Let True Positive (TP) represent the number of correctly identified edges, True Negative (TN) represent the number of correctly identified missing edges, False Positive (FP) represent the number of incorrectly identified edges, and False Negative (FN) represent the number of incorrectly identified missing edges across simulations. Therefore, the total number of ground-truth missing edges is FP+TN, and the total number of ground-truth existing edges is TP+FN. IFPR is defined as: $\text{IFPR}=(1-\frac{\text{FP}}{\text{FP+TN}})\cdot 100,$ which measures the ratio of the number of correctly identified missing edges by the algorithm to the total number of true missing edges. Note that the rate is reported such that 100% corresponds to no falsely detected edges. TPR is defined $\text{TPR}=(\frac{\text{TP}}{\text{TP}+\text{FP}})\cdot 100$ as the ratio of the number of correctly identified edges by the algorithm to the total number of true edges in percent. The Combined Score (CS) is given by Youden’s Index [38, 39], as follows, CS = TPR−FPR.

**Fig 3:**
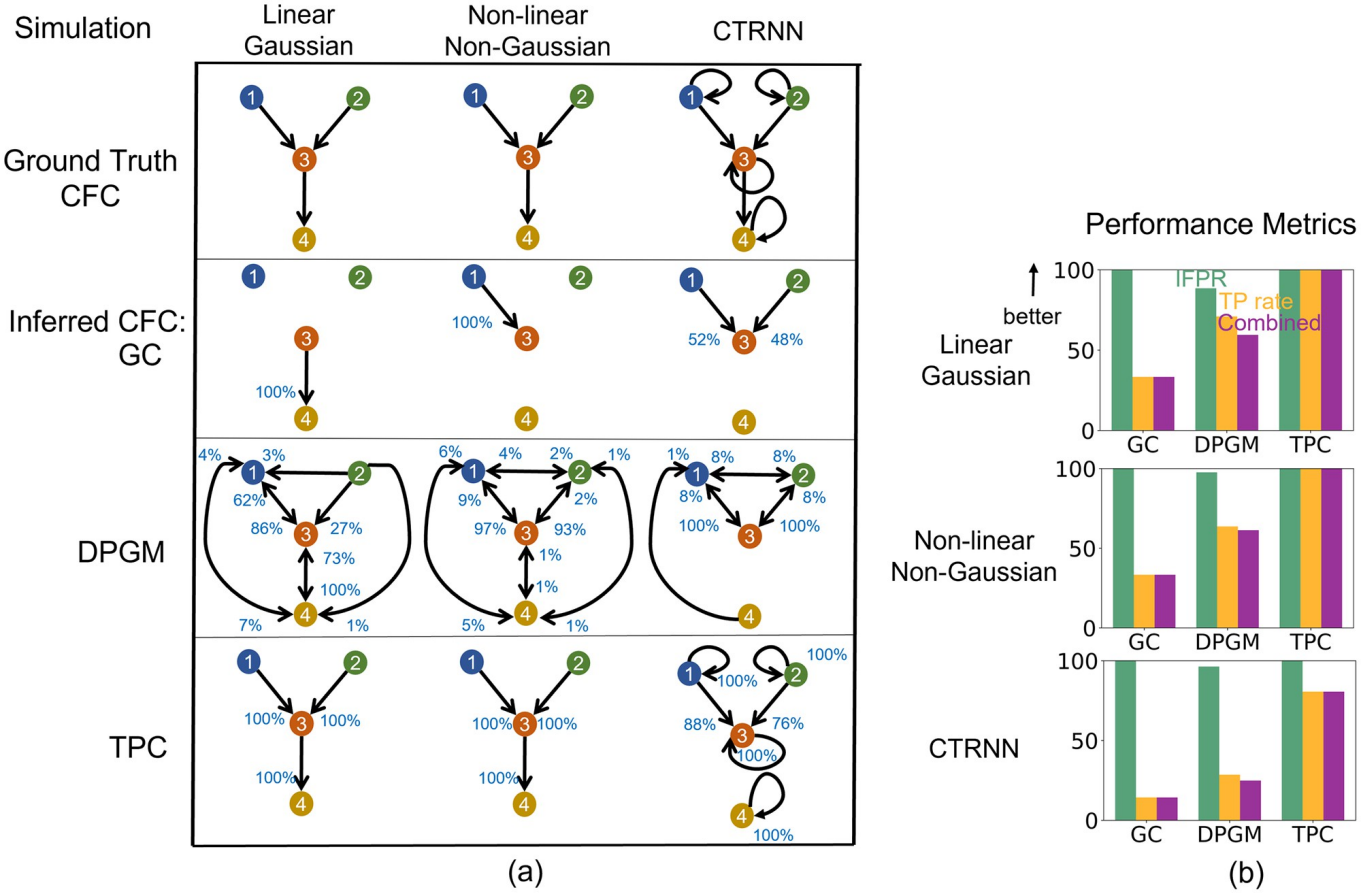
Comparative study of CFC inference. (a) CFC inference by GC, DPGM, and TPC, is compared on three examples of motifs and simulation paradigms; from left to right: Linear Gaussian, Non-linear Non-Gaussian, CTRNN. Table: 4-neurons motifs that define the Ground Truth CFC (row 1) are depicted along with inferred CFC over several simulation instances according to the three different methods (row 2–4). Each inferred CFC has an edge *v* → *w* that corresponds to an edge detected in any of the inference instances. The percentage (blue) next to each edge indicates the number of times the edge was detected out of all instances. (b) IFPR (green), TP rate (orange) and Combined Score (purple) of each method are shown for each motif.

**Fig 4:**
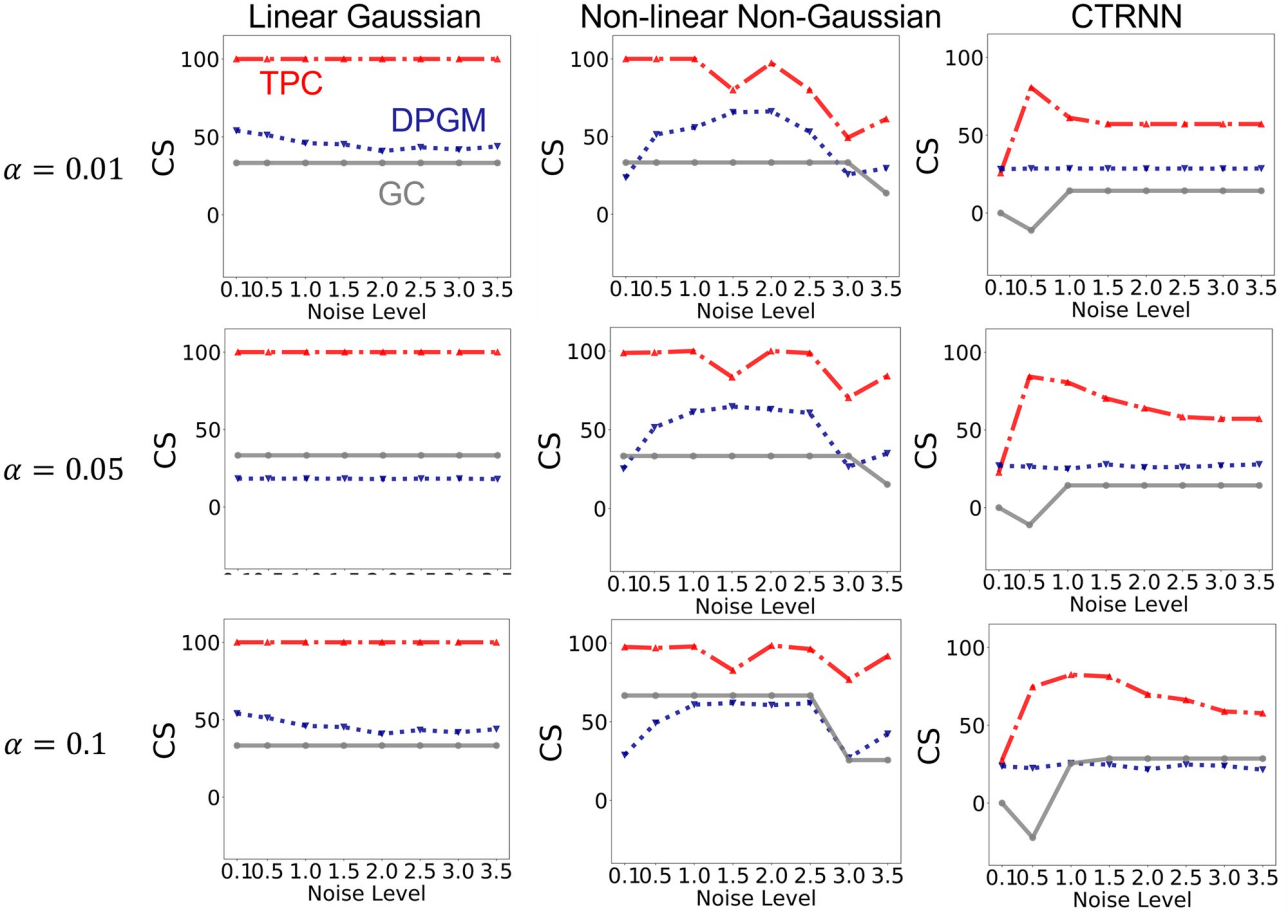
Comparative study over levels of noise and thresholding parameter. Combined Score of the three methods of CFC inference—TPC (red), DPGM (blue), GC (gray), over varying noise levels in simulation *η* = 0.1, 0.5, 1.0, …, 3.5, for simulated motifs from Linear Gaussian, Non-linear Non-Gaussian and CTRNN paradigms (left to right), with thresholding parameter *α* = 0.01, 0.05, 0.1 (top to bottom).

In the motifs and simulation paradigms that we consider, there are 4 neurons and 16 possible edges (including self-loops) per simulation resulting with total of 400 possible edges across 25 simulations. Fig 3 compares in detail the results for GC, DPGM and TPC in inference of true CFC for noise level *η* = 1 and thresholding parameter *α* = 0.05. Here we also report the percentage of the simulations that has each estimated edge present. Higher percentage indicates higher confidence in the detection of that edge. Fig 4 compares the Combined Score of the approaches over different values of noise level *η* and thresholding parameter *α* for each simulation paradigm.

In *Linear Gaussian scenario (left column in* Fig 3), the connections between neurons in the Ground Truth CFC are excitatory due to positive coefficients in the linear dynamical equation for neural activity. GC generates a sparse set of edges in which it correctly detects a single edge 3 → 4 among the three edges of the true CFC but misses two other edges. DPGM generates a large number of edges (9 out of 16), many of which are spurious, though it has a high percentage for expected edges in the Ground Truth CFC (1 → 3, 3 → 4 with 87% and 100% respectively). TPC obtains the Ground Truth CFC, with no spurious edges and obtains the expected edges in all of the trials (1 → 3, 2 → 3, 3 → 4 with 100%, 100% and 100% respectively). Overall, GC, DPGM and TPC produce IFPR = 100%, 88.5%, 100%, TPR = 33.3%, 71.0%, and 100%, and CS = 33%, 59%, 100% respectively. Thereby, among the three methods, we conclude that TPC detects the edges perfectly, while GC is highly specific to correct edges, but since it does not detect two out of three edges it is not as sensitive as DPGM.

In the *Non-linear Non-Gaussian scenario (second column)*, in the Ground Truth CFC consists of 1 → 3, 3 → 4 excitatory due to sin(*x*) being an increasing function, while 2 → 3 is an inhibitory connection due to cos(*x*) being a decreasing function for *x* ∈ [0, 1] in the dynamical equation. As previously, GC consistently detects a sparse set of edges (single edge 1 → 3 with 100%) which is one of the three true edges. DPGM again generates a large number of edges, some of which are spurious. In the majority of trials (97% and 93%, respectively), DPGM correctly obtains two of the three true edges 1 → 3 and 2 → 3. In contrast, TPC obtains no spurious edges and the true edges were detected for all the trials (1 → 3, 2 → 3,3 → 4 with 100%, 100%, 100%). In summary, GC, DPGM and TPC yielded IFPR = 100%, 97.6%, 100% and TPR = 33.3%, 63.7%, 100% and CS = 33%, 61%, 100%. For this scenario, TPC again has the highest performance among the methods.

In *CTRNN scenario (third column)*, self-loops are present for each neuron, and due to positive weights and increasing activation function *σ*(⋅) in their dynamical equation, the connections in the Ground Truth CFC are excitatory. GC obtains two of the three true non-self edges 1 → 3, 2 → 3 for 52%, 48% of the trials. DPGM detects spurious edges, but also infers the non-self true edges 1 → 3, 2 → 3 for 100% of the trials. In comparison, TPC infers no spurious edges and all the self true edges for 100% of the trials and non-self true edges 1 → 3 and 2 → 3 for 88%, 76% of the trials. It is noteworthy that none of these approaches report the 3 → 4 edge in the CTRNN setting. This is suspected to be because of the dynamics of the process in which the strength of the self-inhibition of neuron 4 far exceeds the damped influence (via tanh activation function) from neuron 3 to 4. In summary, IFPR of GC, DPGM and TPC is 100%, 96.3%, 100% and TPR is 14.3%, 28.6%, 80.6% and CS is 14%, 25%, 81% respectively. Among all methods, TPC has the highest TPR, followed by DPGM and lastly GC. TPC and GC have the highest IFPR having not detected any false edges, followed by DPGM. In terms of the CS, TPC has the highest performance compared to other methods.

We compare Combined Score of TPC and other approaches across varying levels of simulation noise *η* from 0.1 to 3.5 and thresholding parameter *α* = 0.01, 0.05, 0.1 in Fig 4. In the Linear Gaussian scenario, we note that TPC has a CS of ≈ 100% across all levels of simulation noise and thresholding parameter *α*, and is followed by DPGM in performance and lastly GC. In the Non-linear Non-Gaussian scenario, TPC has the highest CS compared to other methods across levels of noise and *α*. In the CTRNN scenario, the performance of all the three approaches is lower compared to the other simulation paradigms for different level of *η* and *α*, yet TPC has higher CS compared to the other methods over the different parameter values.

**Fig 5:**
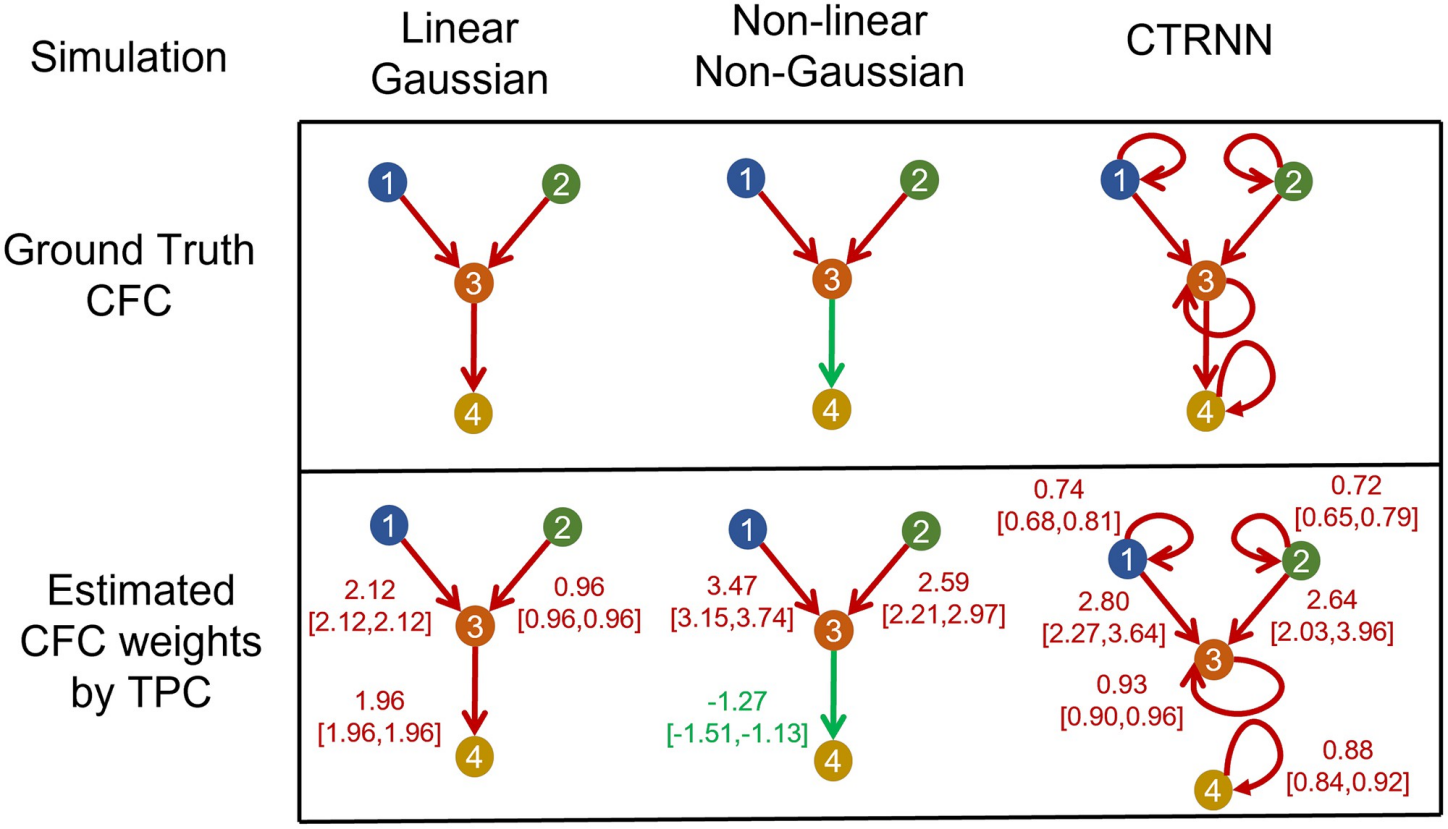
Interventional connectivity weights. Inference of interventional connectivity weights by the TPC algorithm with max delay 1 msec for the example motifs from the three simulation paradigms: Linear Gaussian VAR, Non-linear Non-Gaussian VAR, CTRNN (left to right). Top row: Ground Truth CFC with excitatory (red) and inhibitory (green) connections; Bottom row: Estimated CFC labeled with edge weights (median [min,max] over all instances) and inferred nature whether excitatory (red) or inhibitory (green).

We demonstrate the connectivity weights obtained by TPC and inferred nature of connections, whether excitatory or inhibitory, across simulations for noise level *η* = 1 and thresholding parameter *α* = 0.05 in Fig 5. In the *Linear Gaussian scenario*, the estimated connectivity weight of 1 → 3, 2 → 3, 3 → 4 are 2.12, 0.96, 1.96, across simulation trials. Since the weights are positive, thereby the connections are labeled to be excitatory in all simulation trials, which agrees with the Ground Truth. In the *Non-linear Non-Gaussian scenario*, the estimated weight for the connection 2 → 3 is −1.27 in median and ranges between −1.51, −1.13. Therefore the weight is always negative and labeled inhibitory in the simulation trials. The weight for 1 → 3, 2 → 3 are 3.47, 2.57 in median and ranges between 3.15, 3.74 and 2.21, 2.97 respectively. Their weights are always positive and labeled excitatory in all the simulation trials. These labels for the nature of connections obtained by TPC agrees with the ground truth. In the *CTRNN scenario*, the estimated connectivity weight for 1 → 1, 1 → 3, 2 → 2, 2 → 3, 3 → 3, 4 → 4 are 0.74, 2.80, 0.72, 2.64, 0.93, 0.88 in median respectively and ranges over positive values in all the simulation trials. Thereby the connections are labeled excitatory in all the simulation trials, which agrees with the Ground Truth.

### 3.2 Application to benchmark data

We applied TPC to find the CFC from datasets in the public benchmarking platform—*CauseMe* [40], and compared with benchmarked approaches in the platform in their performance to recover causal interactions present in the datasets. We used the *River Runoff* (real data) and *Logistic Map* (synthetic data) benchmarking datasets (See Section D in S1 Appendix). We compare the approaches PCMCI-GPDC [41], selVAR [42]—which are among the top of the leaderboard for performance on the benchmarking datasets, GC, DPGM (estimated by the PC algorithm), and TPC (Our).

As previously, we measured the performance of the algorithms using 1—False Positive Rate (IFPR), True Positive Rate (TPR) and Combined Score given by Youden’s Index (CS = TPR—FPR) (See Table 1). The River Runoff dataset is comprised of contemporaneous interactions and is expected to demonstrate the performance of the methods in an empirical setting. The Logistic Map dataset is synthetic and excludes contemporaneous interactions and shows the performance of the methods when the ground truth connectivities are specifically controlled for.

**Table 1:** Comparison of CFC inference by GC, DPGM, PCMCI-GPDC and selVAR, and TPC on benchmarking datasets. For each dataset, each method’s Combined Score, True Positive Rate, and 1-False Positive Rate are reported (Higher value is better).

Combined [True, 1-False] Rates (%)		
Algorithm	River-Runoff (Real)	Logistic Map (Synthetic)
GC	37 [45, 92]	79 [86, 93]
PCMCI	45 [100, 45]	86 [89, 97]
selVAR	54 [91, 63]	**87** [88, 99]
DPGM	60 [64, 96]	19 [27, 92]
TPC (Our)	**72(+12%)** [100, 72]	84(-3%) [90, 94]

In terms of CS, TPC has recorded the best performance with a score of 72%, followed by DPGM, selVAR, PCMCI-GPDC and GC at 60%, 54%, 45%, 37% respectively. TPC exceeds the second best approach **by 12%**. In terms of TPR, TPC and PCMCI have the highest scores at 100%, followed by selVAR, DPGM and GC at 91%, 64%, 45% respectively. In terms of IFPR, DPGM has the best performance with a score of 96% closely followed by GC at 92%, TPC at 72%, and selVAR and PCMCI with 63%, 45%. DPGM and GC turn out to have higher IFPR than TPC because they detect fewer false edges, but that is achieved by their detection of fewer edges altogether including fewer true edges leading to a low TPR. In contrast, TPC has greater sensitivity in detecting edges, whose benefit is that TPC detects all the true edges correctly leading to 100% TPR. In terms of both TPR and IFPR, TPC maintains a better trade-off, and thereby a better CS compared to other methods.

For the logistic map dataset, in terms of CS, selVAR, PCMCI-GPDC and TPC have scores of 87%, 86%, 84% respectively, followed by GC with 79% and lastly DPGM with 19%. In terms of TPR, TPC has the highest score of 90%, followed by PCMCI, selVAR, GC and TPC with TPR of 89%, 88%, 86%, 79% respectively, with DPGM having comparatively lowest TPR of 27%. In terms of IFPR, all the approaches have a score of at least 90%. Thereby, TPC achieves a high CS of 84%, short of 3% from the best CS by selVAR of 87%.

The results indicate that in the real benchmark dataset of River-Runoff, TPC outperforms all methods by a substantial gap, whereas, in the synthetic benchmark dataset of Logistic Map, TPC has Combined Score of 84%, being in the top group of 84−87% CS performance. While selVAR and PCMCI achieve a CS of 87% and 86% respectively in the synthetic dataset, they achieve a low CS of 54% and 45% in the real dataset. Since the synthetic dataset is generated by a model controlling coupling strength between variables, low noise and devoid of contemporaneous interactions, thereby most of the methods including TPC perform fairly well in the range of 80%. In contrast, in the real dataset, the coupling between variables as well as noise are not controlled and contemporaneous interactions are expected to be present as the sampling resolution is greater than the time taken for interactions between the variables. Thereby, the real dataset provides a challenge for the methods where TPC outperforms other approaches with a CS and shows significant improvement in performance than the other methods. Presence of TPC in top group of performance for both benchmarks indicates the generality and applicability of TPC to various scenarios.

### 3.3 Application to neurobiological data

We proceed and test the methods on neural data consisting of electrophysiological recordings in the Visual Coding Neuropixels dataset of the Allen Brain Observatory [44, 45]. We compare TPC with Granger Causality (GC) and Sparse Partial Correlation, that are popular methods for obtaining CFC and Associative Functional Connectivity (AFC) from electrophysiological neural recordings. The dataset consists of sorted spike trains and local field potentials recorded simultaneously from up to six cortical visual areas, hippocampus, thalamus, and other adjacent structures of mice, while the mice passively view a stimuli shown to them. The stimuli include static gratings, drifting gratings, natural scenes/images and natural movies, which are shown to the mice with repetitions. The data has been recorded from the neurons with the recently developed technology of Neuropixels which allows real-time recording from hundreds of neurons across the brain simultaneously by inserting multiple probes into the brain [46]. Details of the dataset are in Section E in S1 Appendix.

**Fig 6:**
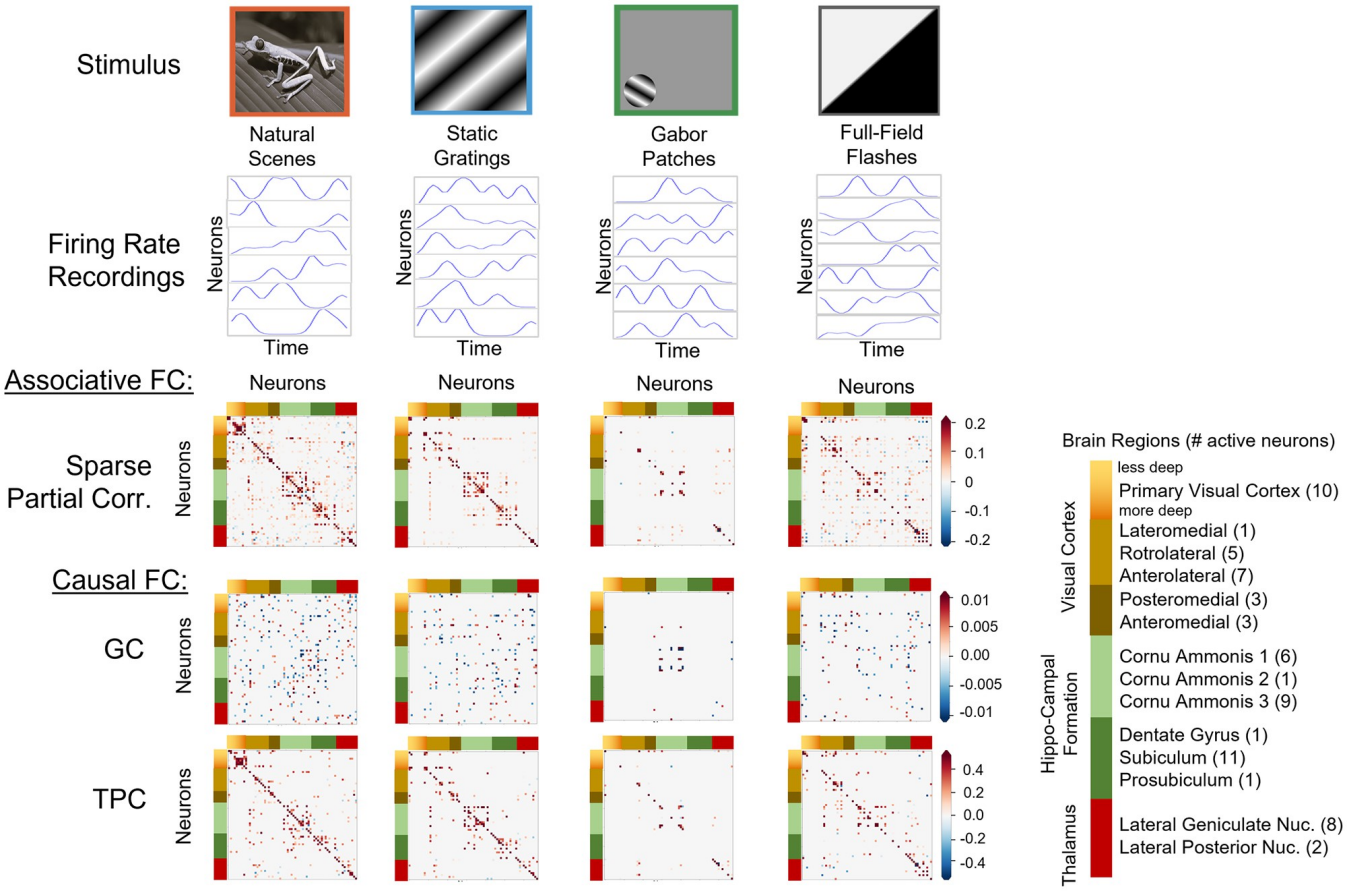
Application to Neuropixels dataset. Comparison and demonstration of the FC inferred for a benchmark of mice brain data from the Allen Institute’s Neuropixels dataset, by three methods for FC inference: Associative FC using Sparse Partial Correlation, and Causal FC using GC and TPC. The estimated FC is represented by its adjacency matrix with edge weights, which is symmetric for Associative FC and asymmetric for Causal FC. The mice were subject to different stimuli, among which we selected four stimuli categories with distinct characteristics: Natural Scenes, Static Gratings, Gabor Patches and Full-Field Flashes [43]. The neurons are clustered by the region of brain: Visual Cortex, Hippo-Campal Formation, and Thalamus, which are further divided into sub-regions. In the adjacency matrices, a non-zero entry in (*i*, *j*) represents the connection of neuron *i* → *j*.

Fig 6 shows the adjacency matrices for the FC obtained by the methods for one trial in each of the stimuli categories. For each stimuli categories, the AFC constitutes a distinct pattern of connectivity among the neurons. We quantify the similarity between a pair of adjacency matrices *A* and *B* by element-wise Pearson’s correlation between *A* and *B*, i.e. Pearson’s correlation between the vectorized form of the matrices *A* and *B*. The CFC obtained by GC has a correlation of -0.02, -0.01, -0.03, -0.02 with the AFC for natural scenes, static gratings, Gabor patches and flashes respectively. In contrast, the CFC obtained by TPC has a correlation of 0.89, 0.88, 0.92, 0.90 with the AFC for natural scenes, static gratings, Gabor patches, and flashes respectively. It is expected that the CFC will be a directed subgraph of the AFC and be consistent with the overall patterns present in the AFC [47, 48]. However, the patterns present in the CFC obtained by GC do not match with the AFC as indicated by low element-wise correlations between the matrices. In contrast, the overall patterns present in the CFC obtained by TPC indeed match with the AFC as indicated by high element-wise correlation between the matrices. On a detailed level, there are differences between TPC-CFC and AFC: TPC results in a directed graph thereby its adjacency matrix is asymmetric while AFC is an undirected graph with symmetric adjacency matrix. Furthermore, the CFC obtained by TPC includes self-loops represented by the diagonals of the adjacency matrix and results in a sparse matrix devoid of noise since the connections passed conditional independence tests and bootstrap stability thresholds. In the CFC obtained by TPC (see Fig 6), a greater extent of connectivity within the active neurons in Primary Visual Cortex is evoked by natural scenes, in Posteromedial and Anteromedial Visual Cortex by static gratings, in Anterolateral Visual Cortex and Thalamus by full-field flashes, compared to other stimuli. All four stimuli exhibit distinct patterns of connectivity in the Cornu Ammonis regions of the Hippo-Campal Formation. Natural Scenes and Static gratings evoke more prominent connectivity within the Subiculum compared to other stimuli.

#### 3.3.1 Graphical comparison of estimated CFC over stimuli

To study the differences in functional connectivity between the stimuli categories, we investigate the topological patterns in the CFC estimated above. The topological patterns can be summarized by graph theoretic measures [49–51], as follows. The graph measures were computed using the *Networkx* Python library [52], over different trials of each stimuli.

- Betweenness centrality: the fraction of all shortest paths that pass through a node, averaged over nodes, indicating the average effect of individual nodes on information flow among the remaining network’s nodes.
- Transitivity: the fraction of all possible triangles present in the graph, indicating prevalence of clustered connectivity.
- Assortativity: measures the similarity of connections in the graph with respect to the node degree.
- Clustering Coefficient: the average of all clustering coefficients in the network, reflecting the tightness of connections between nodes.
- Global Efficiency: average inverse shortest path length, reflecting node’s ability to propagate information with other nodes in the graph.
- Local Efficiency: measures the global efficiency for the neighborhood of a node, averaged over nodes, indicating efficiency of transmitting information by nodes with their neighborhood in the graph.

**Fig 7:**
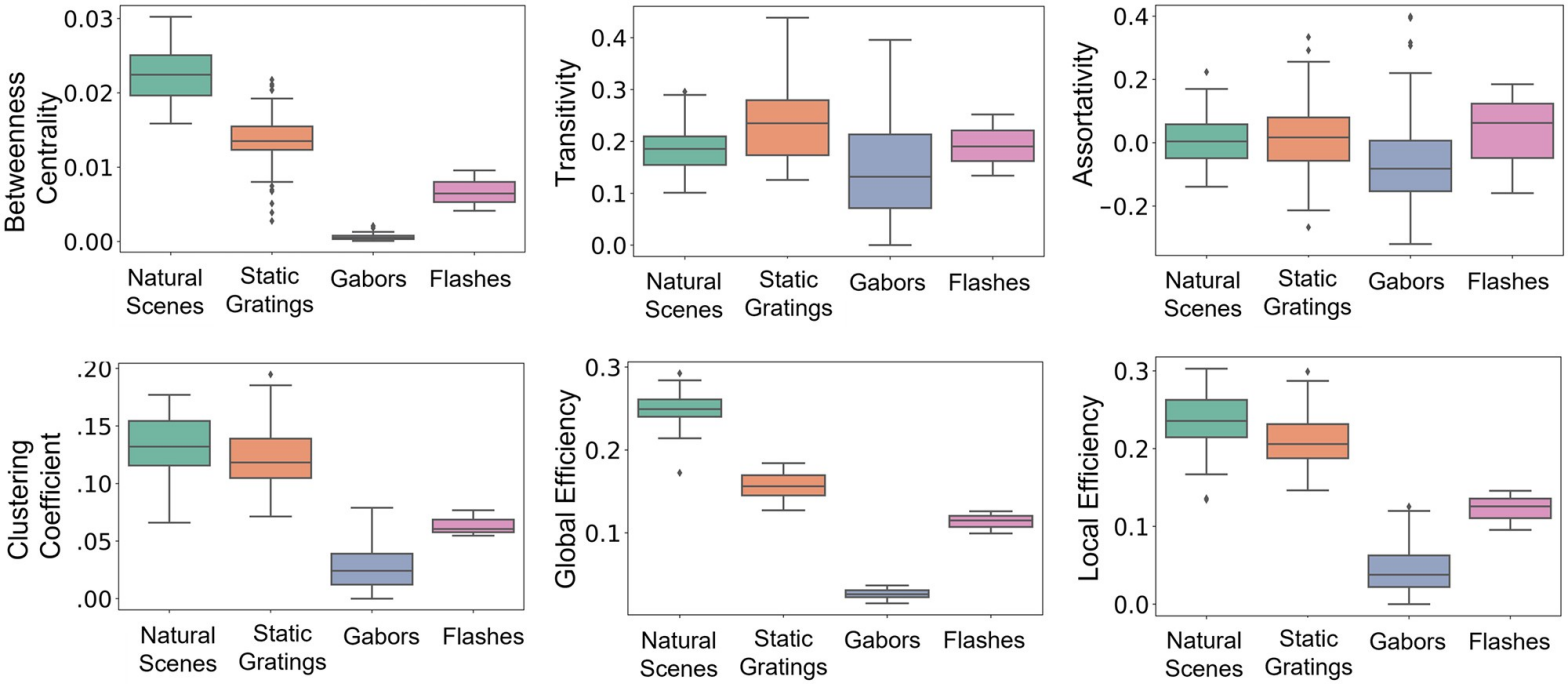
Graphical comparison of estimated CFC over stimuli. This figure compares the distribution of graph measures of CFC obtained by TPC over different stimuli: natural scenes, static gratings, Gabor patches and flashes. The distribution for each graph measure and stimuli is shown by a boxplot.

The results of the graph measures for different stimuli are summarized by boxplots. The boxplot of a graph measure (e.g. betweenness centrality) for a stimulus (e.g. natural scenes) shows the distribution of the values of the graph measure over trials for that stimulus, with the top and bottom of the box indicating the upper and lower quartiles of the distribution, the middle of the box indicating the median, while the whiskers extend to show the rest of the distribution excluding outliers, which are marked by points (See Fig 7).

In Fig 7, in terms of *betweenness centrality*, natural scenes have higher score compared to other stimuli, followed by static gratings, then flashes and lastly Gabor patches. This shows that during natural scenes, the active neurons have a more remarkable effect on the neural information flow, compared to other stimuli, while Gabor patches have the least remarkable effect. In terms of *transitivity*, the scores between the stimuli are close, though static gratings have a relatively higher transitivity, followed by natural scenes, flashes and Gabors. This indicates that static gratings evoked a relatively higher prevalence of clustered connectivity, followed by natural scenes, flashes and lastly Gabor patches. In terms of *assortativity*, the stimuli did not evoke a comparatively distinct score as well. In terms of *clustering coefficient*, natural scenes and static gratings have higher scores compared to flashes and Gabors. This shows that, natural scenes and static gratings have the most tightness of connections between nodes in the graph, flashes have a comparatively lower tightness of connections, while Gabor patches have the least score. In terms of *global efficiency*, natural scenes have the highest score, while, static gratings have comparatively lower score, followed by flashes and Gabor patches. This shows that natural scenes evoked comparatively highest efficiency of information propagation in the CFC globally, followed by static gratings, flashes and Gabors. In terms of *local efficiency*, the trend across stimuli is similar to global efficiency, while natural scenes and static gratings evoked a more similar local efficiency compared to global efficiency. This shows that the efficiency in information propagation in local neighborhoods of neurons is higher for natural scenes and static gratings to a similar extent, but with higher efficiency compared to flashes and Gabor patches. In conclusion, betweenness centrality, clustering coefficient, global and local efficiency indicate a differential involvement of brain regions for different stimulus types.

It is noteworthy that while transitivity and assortativity are measures found on the graph as a whole, the remaining measures are found for each node and then averaged upon the nodes of the graph. So, transitivity and assortativity gives a high weight upon nodes with a high degree while the other measures give comparatively greater weight upon nodes with a low degree. This can explain the different pattern seen for transitivity and assortativity compared to the other measures in Fig 7. It is also implied that the greater weight for low degree nodes leads to a different distribution of the measures of betweenness centrality, clustering coefficient, global and local efficiency across different stimulus types, in contrast to similar measures for transitivity and assortativity across different stimulus types.

## 4 Discussion

In this paper, we propose a novel methodology, the TPC Algorithm, for finding causal functional connectivity between neurons from neural time series using Directed Probabilistic Graphical Models (DPGM). In particular, we extend the applicability of DPGM to CFC inference from time series by unrolling and implementing the Directed Markov Property (DMP) to obtain the unrolled DAG reflecting causal spatial and temporal interactions. We then roll the DAG back to obtain the CFC graph. The methodology exhibits interpretability of causal interactions over time between neural entities. It also incorporates time delays in interactions between neurons as well as the presence of feedback-loops. The model and the approach are non-parametric, meaning that it does not require the specification of a parametric dynamical equation for neural activity. We show that if the neural activity obeys an arbitrary dynamical process, the Rolled CFC-DPGM is consistent with respect to the causal relationships implied by the dynamical process. We determine that the Rolled CFC-DPGM is predictive of counterfactual queries such as ablation or modulation. We show that the answers can be provided by using simple causal reasoning with the edges of the rolled CFC-DPGM. We demonstrate the utilization of the methodology in obtaining CFC from simulations and compare the performance of TPC with other methods such as Granger Causality (GC) and common DPGM. Furthermore, we apply the methods to benchmarks of time-series causal inference and neurobiological dataset from mice brain presented with various visual stimuli. The results provide insights into the CFC between neurons in the mouse brain in a variety of stimuli scenario. We also compare the topological patterns in the estimated CFC between different stimuli using graph-theoretic measures.

The distinctive and useful aspect of TPC is that it takes into consideration neural interactions over time. In neuroscience literature, causality is typically referred to as “a cause of an observed neural event (the ‘effect’) as a preceding neural event whose occurrence is necessary to observe the effect” [2]. The approach of unrolling causal graphs over time and considering time-delays in TPC incorporates this definition by essentially finding whether the previous time values of the neurons impact the present value of a particular neuron by the obtained directed graph. By virtue of the Directed Markov Property, TPC incorporates causality of neural interactions in a non-parameteric, model-free manner and incorporates interventional properties. The bootstrapping step filters spurious connections by repeating the inference of CFC over several subsampled blocks of the time series and discarding those connections that are absent in multiple repetitions. Pruning further selects edges by discarding edges with exceedingly low edge weight. In contrast, parametric approaches, such as GC, do not satisfy the Directed Markov Property, thereby not incorporating interventional properties. Furthermore, in comparative studies with simulated, benchmark and real neurobiological datasets, we found the performance of TPC to be better compared to other approaches. We conclude by summarizing, in Table 2, the differences and benefits of TPC in a comparison with other approaches including variants of the PC algorithm (DPGM) which satisfy DMP in a static data setting, and other approaches that do not obey the DMP, such as Granger Causality (GC) and Dynamic Causal Model (DCM), outlining their strengths and weaknesses with respect to several criteria of causality in functional connectomics. Indeed, capturing as many causal criteria is fundamental to any approach from statistical and application points of view.

**Table 2:** Comparative summary of different approaches for causal modeling.

	GC	DCM	DPGM	TPC
Form of Causality	Non-zero parameters in VAR model	Coupling parameters in biological model	Directed Markov Graph	Directed Markov Graph over time-advanced variables
Inclusion of temporal relationships	**Yes**	**Yes**	**No**, formulation for static variables	**Yes**, adapts DPGM for inter-temporal relationships
Inclusion of contemporaneous relationships	**No**	**Yes**, by a differential equation	**No**	**Yes**, if (*i*, *t*) → (*j*, *t*) then *i* → *j*.
Generalizable Statistical Model	**Yes**	**No**	**Yes**	**Yes**
Non-parametric Model	**Yes**, parametric and non-parametric approaches exist.	**No**, biologically mechanistic non-linear model.	**Yes**, equivalent to an arbitrary functional relationship between nodes.	**Yes**, equivalent to an arbitrary functional relationship between neural activity at different times.
Supports CFC Inference	**Yes**	**No**, suitable for comparing model hypotheses	**Yes**	**Yes**
Cycles (including self-loops) Occuring In The Model	**Yes** for VAR model (neuron *i* → *i* when *A*_*ii*_(*k*) ≠ 0 for some *k*).	**Yes** (*i* → *i* when *θ*_*ii*_ ≠ 0)	**No**, it is a DAG	**Yes** (*i* → *i* when (*i*, *t*) → (*i*, *t*′) for some *t* < *t*′)
Incorporation of Interventional and Counterfactual queries	**No**	**No**	**Yes** but for static variables.	**Yes**, adapts for temporal scenario, can predict the consequence on CFC of counterfactual intervention to neural activity.

Our exposition of properties of each approach and the comparative study show that each of the methods address different aspects of modeling causality of neural interaction and mapping them in the form of a graph [6]. The comparative table demonstrates that with respect to the model that each approach is assuming, GC requires a linear model in its common use, though recent non-linear and non-parametric extensions, have been applied. DCM requires a strict well defined mechanistic biological model and thus can only compare different models based on evidence from data. In comparison, DPGM and TPC have the advantage of not requiring modeling of the neural dynamics using a parametric equation or assumption of a linear model. While DPGM is developed for static variables, and as such cannot address temporal and contemporaneous relationships and must obey DAG architechture, TPC is suited for time series setting and extends DPGM for spatiotemporal data. TPC obtains the CFC that follows Directed Markov Property extended to include inter-temporal relationships in a time-series setting such that parent-child relations between neurons are equivalent to arbitrary functional relationships between their neural activity over time. In terms of incorporating contemporaneous interactions arising when causal interactions happen faster than sampling rate, while GC and DPGM do not, TPC is able to incorporate contemporaneous interactions by design. In terms of incorporating self-loops in neural activity, while DPGM typically produces a DAG, TPC incorporates self-loops in neural activity. In regards to guarantee of causality, GC can provide useful insights into a system’s dynamical interactions in different conditions, however its causal interpretation is not guaranteed as it focuses on the predictability of future based on past observations of variables. DCM uses the parameters for coupling between hidden neural states in competing biological models to indicate CFC, however it compares hypothetical models based on evidence from data which relevance to causality is not guaranteed (Friston et al., 2003). In summary, TPC extends DPGM to the time-series setting and provides a probabilistic foundation for causality which is predictive of the consequence of possible intervention like neuron ablation and neuromodulation.

While the unrolled graphical modeling can capture contemporaneous interactions over distinct neurons, it does not capture contemporaneous self-interactions for the same neuron. This is due to the requirement of DPGM for the unrolled graphical model to be a directed acyclic graph. Although this is a limitation, it is still an improvement from Granger Causality for which the VAR model does not allow any contemporaneous relations [53].

While TPC provides a powerful causal framework for time series, its current version relies on the PC algorithm which in turn assumes *causal sufficiency*, that is, all the causes of the input variables are present within the input variables. In terms of neurons, causal sufficiency means that neurons that have a causal influence on the observed neurons are also observed. There is no other requirement on the number of neurons. However, causal sufficiency in real neural data is challenging to achieve since it is often the case that there are uncaptured neurons which may causally influence the observed neurons [32, 54]. In the absence of causal sufficiency, Step 3 of TPC involving PC algorithm would estimate the true causal edges between the observed neurons but also a spurious edge between a pair of observed neurons whenever an unobserved neuron is a common cause to both of the observed neurons in the pair [55, 56]. These are partly remediated by the TPC algorithm by discarding spurious edges in the Bootstrap and Pruning steps, but some of spurious edges may remain. To address this, an alternative strategy that could be considered is replacing the PC algorithm with the FCI algorithm in Step 3 of TPC (1) since the FCI algorithm exhibits statistical consistency without causal sufficiency, that converges in probability to the true causal relationships, given i.i.d. samples [57]. However, the FCI algorithm identifies indirect connections only and not direct connections, thereby would result in a CFC with indirect causal connections.

Under the assumptions of 1) causal sufficiency, 2) faithfulness, and 3) given i.i.d. samples, the PC algorithm’s estimated causal DAG is statistically consistent. In light of these assumptions, the step 2 of the TPC algorithm constructs samples with a time-advance of 2(*τ* + 1) between samples from a bootstrap window, for max time-delay of interaction *τ*, which are used as an input to the PC algorithm in the next step. The time-gap between samples of 2(*τ*+ 1) reduces between-sample dependence. And, the samples being constructed from a short bootstrap window instead of the entire time series aids to make their distribution fairly identical. Yet, specifications of *τ* of lower value could lead to between-sample dependence and rapid perturbations to the time series in a bootstrap window can lead to distribution changes between samples in a bootstrap window, leading to potential reduction in efficacy of edge detection by the PC algorithm. To improve overall efficacy in such scenarios and curb spurious edge detections, the bootstrap step of the TPC algorithm outputs many CFCs over random time windows and preserves only stable edges over the set of CFCs. In the pruning step, the edges in the CFC are pruned if having an exceedingly low connectivity weight.

In conclusion, TPC provides a probabilistic and interpretable formulation for CFC modeling and inference in the context of neural time series. We have established the statistical properties of the model as well as demonstrated its performance in estimation of CFC. We have demonstrated TPC application in continuous time series datasets, however TPC is similarly applicable to discrete time series datasets by simply using a statistical conditional independence test for discrete data in the algorithm. This can be especially relevant for count datasets such as spiking neuron datasets.

## 5 Materials and methods

### 5.1 Causal functional connectivity for static variables—Review

In this section, we provide a concise summary of DPGM for finding CFC for static variables, i.e. variables with **i.i.d. samples**, that TPC extends to incorporate temporal dependence in time series. Let us consider a brain network *V* = {*v*_1_, …, *v*_*N*_} with *N* neurons labeled as *v*_1_, …, *v*_*N*_ and $X_{v}\left(t\right)\in R$ denote a random variable measuring the activity of neuron *v* at time *t*. Examples for such variables are instantaneous membrane potential, instantaneous firing rate, etc. Let *Y*_*v*_ denote a scalar-valued random variable corresponding to *v* ∈ *V*, e.g., the neural recording at time *t*: *Y*_*v*_ = *X*_*v*_(*t*), average of recordings over time $Y_{v}=\bar{X}\left(v\right)$, and for a set of neurons *A* ⊂ *V*, ***Y***_*A*_ denotes the random vector (*Y*_*v*_, *v* ∈ *A*). Let *G* = (*V*, *E*) denote a *directed acyclic graph* (DAG), i.e., a directed graph without directed cycles, over the neurons in *V* and with directed edges *E*. Nodes *u* and *v* ∈ *V* are said to be *adjacent* if *v* → *u* ∈ *E* or *u* → *v* ∈ *E*. A *path* is a sequence of distinct nodes in which successive nodes are adjacent. For a path *π* = (*v*_0_, …, *v*_*k*_), if every edge of *π* is of the form *v*_*i*−1_ → *v*_*i*_ then *v*_0_ is an *ancestor* of *v*_*k*_ and *v*_*k*_ is a *descendant* of *v*_0_. The set of *non-descendants* of *v*, denoted *nd*_*G*_(*v*), contains nodes *u* ∈ *V* \ {*v*} that are not descendants of *v*. The set of *parents* of *v* ∈ *V* is denoted as *pa*_*G*_(*v*) = {*u* ∈ *V* : *u* → *v* ∈ *E*}. We mark the set *nd*_*G*_(*v*)\*pa*_*G*_(*v*) as the set that contains all nodes which are older ancestors of *v* before its parents [8, 9].

With these notations, we highlight the *Directed Markov Property* (DMP) and its functional equivalence for DPGM. The DMP connects probabilistic conditional independencies between nodes of a directed graph with relationships of causal influence by ensuring that the influence of each node’s ancestors beyond parents reaches to the node exclusively via its parents. Furthermore, the functional equivalence for DPGM shows that, the edges in a DPGM satisfying the DMP are consistent with causal functional interactions among the nodes.

#### Directed Markov Property (DMP)

(*Y*_*v*_, *v* ∈ *V*) is said to satisfy the *Directed Markov Property* with respect to the DAG *G* if and only if,

$$\begin{array}{c} Y_{v}⫫Y_{nd_{G}\left(v\right)\backslash pa_{G}\left(v\right)}|Y_{pa_{G}\left(v\right)} \end{array}$$ (1)

The DMP translates the edges in the DAG into conditional independencies, such that for each node *v* and its older ancestors *nd*_*G*_(*v*)\*pa*_*G*_(*v*),*Y*_*v*_ is conditionally independent of $Y_{nd_{G}\left(v\right)\backslash pa_{G}\left(v\right)}$ given the variables corresponding to the parents of *v*, $Y_{pa_{G}}\left(v\right)$. The DMP can be equivalently represented with functional relationships between parent and child instead of conditional independencies, which is described in the following theorem [58].

#### Functional Equivalence of DMP

If *Y*_*v*_ satisfies

$$\begin{array}{c} Y_{v}=g_{v}\left(Y_{pa_{G}\left(v\right)},\epsilon_{v}\right),v\in V \end{array}$$ (2)

where *ϵ*_*v*_ are independent random variables, and *g*_*v*_ are measurable functions for nodes *v* ∈ *V*, then *Y*_*v*_, *v* ∈ *V* satisfies the Directed Markov Property with respect to the DAG *G*. Conversely, if *Y*_*v*_, *v* ∈ *V* satisfies the Directed Markov Property with respect to *G*, then there are independent random variables *ϵ*_*v*_ and measurable functions *g*_*v*_ for which Eq (2) holds. This shows that if *Y*_*v*_, *v* ∈ *V* satisfies the DMP with respect to the DAG *G*, then *G* admits a natural causal interpretation, due to its functional equivalence: parent nodes of *v* in *G* causally influence the child node *v* [59].

#### Peter & Clark (PC) algorithm

Let *Y*_*v*_, *v* ∈ *V* satisfy the DMP with respect to the DAG *G*. The PC algorithm is a popular method to infer *G* from observed data [22]. The PC algorithm uses a consistent statistical test, such as Fisher’s Z-transform when *Y*_*v*_, *v* ∈ *V* are Gaussian variables, and kernel and distance based tests for non-Gaussian variables [60, 61]. The algorithm first represents the observed variables by nodes of a graph and starts with an empty set of edges and puts an undirected edge between each pair of nodes if they are not independent or conditionally independent given any other variable(s) determined by the statistical test. This results in the undirected skeleton graph, which is then converted into a DAG by directing the undirected edges using rules for orientation. The PC algorithm estimates several DAGs ${\hat{G}}_{i}$ based on i.i.d. samples of *Y*_*v*_, *v* ∈ *V*, and outputs a single completed partially directed acyclic graph (CPDAG) $\hat{G}$ defined as follows: $\hat{G}$ has a directed edge from node *v* → *w* if *v* → *w* is present in all the DAGs ${\hat{G}}_{i}$. It has an undirected edge between *v* and *w* if either directions between them are present among the DAGs ${\hat{G}}_{i}$. It has no edge between *v* and *w* if no edge is present between them in any of the DAGs ${\hat{G}}_{i}$. The CPDAG $\hat{G}$ is uniquely identifiable from observed data.

The PC algorithm assumes *causal sufficiency* of the input variables: A set *V* of variables is causally sufficient for a population if and only if in the population every common cause of any two or more variables in *V* is in *V*, or has the same value for all units in the population. Another method, the FCI algorithm, is applicable when causal sufficiency does not hold [57]. However, while the PC algorithm can identify direct causes, FCI algorithm cannot distinguish between direct and indirect causes.

Let *P* denote the probability distribution of *Y*_*v*_, *v* ∈ *V*. The PC algorithm also assumes *faithfulness* of the DAG *G* to *Y*_*v*_, *v* ∈ *V*: if the DMP with respect to *G* encompasses all the conditional independence relations due to *P*, *G* is said to be *faithful* to *Y*_*v*_, *v* ∈ *V*. Using a consistent statistical test for conditional independence, and assuming causal sufficiency and faithfulness, the PC algorithm estimate, $\hat{G}$, is consistent for *G*; that is, $\hat{G}$ converges in probability to *G* with increasing number of samples in data [22, 62].

### 5.2 Choice of gap between time-advanced samples in TPC algorithm

The TPC algorithm considers a choice of 2(*τ* + 1) for gap between time-advanced samples to help reduce between-sample dependence for the time-advanced samples. To justify the choice of 2(*τ* + 1), let the first two time advanced samples with a gap of *r* be $\chi_{0}=\left(X_{0},...,X_{\tau }\right)$ and $\chi_{1}=\left(X_{r},...,X_{\tau +r}\right)$. If *r* < 2(*τ* + 1) then *r* − *τ* ≤ *τ*, and so time *t* = *τ* and time *t* = *r* are within the maximum time delay of interaction, thereby ***X***_*τ*_ and ***X***_*r*_ can have a dependence between them, which implies a dependence between $\chi_{0}$ and $\chi_{1}$. If *r* ≥ 2(*τ* + 1) then *r* − *τ* > *τ* so time *t* = *τ* and time *t* = *r* have a gap greater than the maximum delay of interaction *τ*, therefore, ***X***_*τ*_ and ***X***_*r*_ will be independent. This justifies the choice for the gap *r* to be 2(*τ* + 1).

### 5.3 Connectivity weights in the CFC for time series

In this section, we define the connectivity weights obtained by TPC. Connectivity weights refer to a weight *w*_*uv*_ for connections *u* → *v* in the CFC graph. We consider *interventional causal effects* to define connectivity weights. Interventional causal effects quantify how much effect an intervention applied to neuron *u* will have on neuron *v*.

**Definition 2: Interventional causal effects in Unrolled DAG**. Let ***X*** satisfy the Directed Markov Property with respect to ***G*** = (***V***, ***E***). The *interventional causal effect* of *X*_*u*_(*t*) = *x*_*u*,*t*_ on *X*_*v*_(*t*′), where *x*_*u*,*t*_ are fixed values, for *u*, *v* ∈ *V*, and *t*, *t*′ ∈ {0, 1, …, *T*} with (*u*, *t*) ≠ (*v*, *t*′), is defined in interventional calculus by Pearl et. al as follows [23, 37, 63]

$$\begin{array}{c} \frac{\partial }{\partial x}E\left(X_{v}\left(t^{\prime }\right)|X_{u}\left(t\right)=x\right){|}_{x=x_{u,t}} \end{array}$$ (3)

where, for a random variable *Y*, *E*(*Y*) denotes the expectation of *Y*.

Assuming *X*_*u*_(*t*), *u* ∈ *V*, *t* ∈ {0, 1, …, *T*} are jointly Gaussian, the causal effect does not depend on the value of *x*_*u*,*t*_, and the causal effect of *X*_*u*_(*t*) on *X*_*v*_(*t*′) from Eq (3) takes the following form,

$$\begin{array}{c} w_{u,t}^{v,t^{\prime }}=\{\begin{array}{cc} 0, & \;\text{if}\;\left(v,t^{\prime }\right)\in pa_{G}\left(\left(u,t\right)\right),\\ \text{coefficient}\;\text{of}\;X_{u}\left(t\right)\;\text{in}\;X_{v}\left(t^{\prime }\right)∼X_{u}\left(t\right)+X_{pa_{G}\left(\left(u,t\right)\right)} & \;\text{if}\;\left(v,t^{\prime }\right)\notin pa_{G}\left(\left(u,t\right)\right) \end{array} \end{array}$$ (4)

where $X_{v}\left(t^{\prime }\right)∼X_{u}\left(t\right)+X_{pa_{G}\left(\left(u,t\right)\right)}$ is shorthand for linear regression of *X*_*v*_(*t*′) on *X*_*u*_(*t*) and $X_{pa_{G}\left(\left(u,t\right)\right)}=\left\{X_{a}\left(b\right):\left(a,b\right)\in pa_{G}\left(\left(u,t\right)\right)\right\}$. Note that (*u*, *t*) is a node of G so $pa_{G}\left(\left(u,t\right)\right)$ denotes the set of parents of (*u*, *t*) in G as defined in Section 5.1.

Note that when ***X*** satisfy the Directed Markov Property with respect to ***G*** = (***V***, ***E***), the causal effects are defined in interventional calculus literature for all pairs of nodes in ***V*** and not that only for those pairs which are adjacent. And, if *u* → *v* ∈ ***E*** then the interventional causal effect from *v* to *u* is 0 [37].

Under the Gaussian assumption, we define the *interventional causal effect* of the activity of neuron *u* at time *t* on the activity of neuron *v* at time *t*′ to be $w_{u,t}^{v,t^{\prime }}$ for *t* < *t*′ where *t*, *t*′ ∈ {0, 1, …, *T*}, *u*, *v* ∈ *V*. Using this, we define weights for the connection from *u* to *v* for *u*, *v* in the rolled CFC-DPGM *F*_*τ*_, following the way *F*_*τ*_ is defined from ***G***.

**Definition 3: Interventional connectivity weights in Rolled CFC-DPGM**. Let ***X*** satisfy DMP with respect to the DAG ***G*** = (***V***, ***E***) and *F*_*τ*_ is the Rolled CFC-DPGM with max delay *τ*. If neurons *u*, *v* are connected as *u* → *v* in *F*_*τ*_, then, the *weight of connection* from neuron *u* to *v* with max delay *τ*, denoted by *w*_*τ*_(*u*, *v*), is defined as the average of the causal effects: $w_{u,t}^{v,t^{\prime }}$ for (*u*, *t*) → (*v*, *t*′) ∈ ***E***, *t* ≤ *t*′ ≤ *t* + *τ*, *t*, *t*′ ∈ {0, 1, …, *T*}.

#### Connectivity weights in TPC Algorithm

After the CFC graph $F_{\tau }^{b}$ is obtained in Step 3–5a in TPC algorithm, the interventional connectivity weights for connections in $F_{\tau }^{b}$ are obtained in Step 5b to define the connectivity weights $w_{\tau }^{b}\left(u,v\right)$ for connections $u\to v\in F_{\tau }^{b}$. Then bootstrapping in Step 6 ensures greater stability of the estimated connectivity weights. Step 6 outputs a single connectivity weight *w*_*τ*_(*u*, *v*) for connections *u* → *v* in *F*_*τ*_, as the average of $\left\{w_{\tau }^{b}\left(u,v\right):u\to v\in F_{\tau },u\to v\in F_{\tau }^{b},b\in 1,\ldots ,m\right\}$ when the set is non-empty and 0 otherwise. Therefore, this finds a connectivity weight for the edge *u* → *v* ∈ *F*_*τ*_ by taking the average of connectivity weight of the edge $u\to v\in F_{\tau }^{b}$ whenever it exists over *b*.

#### Pruning by Connectivity Weights

After the rolled CFC-DPGM and Connectivity Weights have been inferred by the TPC Algorithm, spurious connections can be pruned further in Step 7 of TPC (1), by discarding those connections whose connectivity weight is less than a threshold. For this threshold, we use a factor of 10 of the maximum Connectivity Weight in the rolled CFC-DPGM.

From the interpretation of regression coefficients in Eq (4), a negative connectivity weight (5.3) from neuron *u* → *v* in *F*_*τ*_ indicates an inhibitory connection, in which, increased activity *X*_*u*_(*t*) of the pre-synaptic neuron *u* at time *t* causes subjugation of activity *X*_*v*_(*t*′) of the post-synaptic neuron *v* at a following time *t*′, when activity of the neurons that are causally connected to neuron *u* at time *t*, are kept fixed. In a similar manner, a positive FC weight from neuron *u* → *v* in *F*_*τ*_ indicates an excitatory connection. In this way the strength of the functional connection, which also indicates it’s excitatory and inhibitory nature is learnt from the data.

### 5.4 Properties of Rolled CFC-DPGM

We highlight properties of the Rolled CFC-DPGM, obtained by TPC, in capturing causal relationships in neural dynamics in a non-parametric manner and being predictive of the impact of counterfactual interventions to the neurons in the CFC.

#### Well-defined

The transformation from unrolled DAG ***G*** to Rolled CFC, *F*_*τ*_, is a well-defined function, meaning that starting from the same unrolled DAG ***G*** we will not have multiple possible CFC and there will be a unique CFC *F*_*τ*_.

Proof: By contradiction, consider two distinct CFCs *F*_*τ*,1_ and *F*_*τ*,2_ with *F*_*τ*,1_ ≠ *F*_*τ*,2_ obtained from the unrolled DAG ***G*** = (***V***, ***E***). Since *F*_*τ*,1_ ≠ *F*_*τ*,2_, so ∃*i*, *j* ∈ *V* such that *i* → *j* ∈ *F*_*τ*,1_ but *i* → *j* ∉ *F*_*τ*,2_, where *i*, *j* could be either the same or distinct neurons. Using the definiton 1 of Rolled CFC-DPGM, *i* → *j* ∈ *F*_*τ*,1_ implies that for some 0 ≤ *t*_1_ ≤ *t*_2_ ≤ *t*_1_ + *τ*, (*i*, *t*_1_) → (*j*, *t*_2_) ∈ ***E***. But *i* → *j* ∉ *F*_*τ*,2_ contradicts this as it implies that (*i*, *t*_1_) ↛ (*j*, *t*_2_) ∈ ***E*** for any 0 ≤ *t*_1_ ≤ *t*_2_ ≤ *t*_1_ + *τ*.

#### Non-parametric Causal Relations

The following theorem shows that the CFC given by the model is consistent with the ground truth causal relationships between neural activity at different time without requiring any assumptions on the functional form of the relationships. That is, we show that if past time points of neurons in *A*_*v*_ ⊂ *V* influence the present time point of *v* ∈ *V* by an arbitrary function with independent random noise, then neurons in *A*_*v*_ are connected to the neuron *v* in their Rolled CFC-DPGM. This means that causal relationships among the neurons, in their unknown arbitrary dynamical equation, are accurately represented by the CFC without prior knowledge of the functional form of the relationships. The benefit of Rolled CFC-DPGM is that it uses a non-parametric graphical model for the temporal relationships between neurons and does not assume a parametric equation for the temporal relationships. Furthermore, the Rolled CFC-DPGM provides a framework to answer causal questions related to the consequence of interventions and counterfactuals.

**Theorem 1** (Consistency with Time Series Causal Relations). For neurons *v* ∈ *V* with activity *X*_*v*_(*t*) at time *t* ∈ {0, 1, …, *T*}, if *X*_*v*_(*t*) satisfies

$$\begin{array}{c} X_{v}\left(t\right)=g_{v,t}\left(X_{u_{v,1}}\left(t_{v,1}\right),\ldots ,X_{u_{v,p}}\left(t_{v,p}\right),\epsilon_{v}\left(t\right)\right), \end{array}$$ (5)

for some *p* ≥ 1, nodes *u*_*v*,*i*_ ∈ *V*, times *t*_*v*,*i*_ ∈ {*t* − *τ*, *t* − *τ* + 1, …, *t*} with either *u*_*v*,*i*_ ≠ *v* or *t*_*v*,*i*_ ≠ *t*, *i* ∈ {1, …, *p*}, where *τ* is the maximum time-delay of interaction, and *g*_*v*,*t*_ is a measurable function and *ϵ*_*v*_(*t*) are independent random variables, then the graph *F*_*τ*_ with nodes *V* and parents of *v*, *pa*_*Fτ*_(*v*), given by

$$pa_{F\tau }\left(v\right)=\left\{u_{v,1},\ldots ,u_{v,p}\right\}$$

is the Rolled CFC-DPGM between the neurons in *V*.

*Proof*. See Section A in S1 Appendix.

According to Theorem 1, the edges in the Rolled CFC-DPGM corresponds to actual causal relations in the neural time series. TPC takes steps to estimate the Rolled CFC-DPGM, and reports good performance in simulations in recovering the true CFC in comparison to GC and DPGM.

TPC is a non-parametric approach assuming DMP with respect to the unrolled DAG which is satisfied for arbitrary functional relationships (Theorem 1). Estimation using PC algorithm further assumes the converse of DMP, i.e. faithfulness, which is a generic assumption facilitating causal inference. These are broad assumptions which include arbitrary functional relationships and noise distributions, under which TPC is expected to perform well. Estimation using PC algorithm also assumes causal sufficiency, therefore there is a possibility for unobserved causal influence to lead to erroneous edges being outputted by TPC. This is remediated (to some extent) by excluding spurious edges in the bootstrap step of TPC. Even though a non-parametric approach like TPC is expected to perform well irrespective of parametric model assumptions, parametric approaches can outperform it when the parametric model assumptions are precisely satisfied. For example, selVAR is seen to outperform TPC in the Logistic Map synthetic dataset. Also GC, which is a parametric model-based approach, assuming a stationary Gaussian vector autoregressive (VAR) model, expected to perform well when its model assumptions are satisfied. However, irrespective of whether the Gaussian VAR model assumption is satisfied or not, TPC is seen to perform at par with or better than GC in simulations. DPGM is expected to excel in performance in i.i.d. scenarios but would not be generally applicable in capturing functional relationships across-time which require setting *τ* in TPC to be greater than zero.

#### Interventional Properties

The Rolled CFC-DPGM can answer questions concerning counterfactual interventions on neurons without experimentally performing the interventions, i) Ablation of a neuron *A*, ii) Activity of neuron *B* is externally modulated. In the following corollary we show how conclusions can be drawn for such queries.

**Corollary 5.1** (Intervention). For neurons *v* ∈ *V* following the dynamics in equation Eq (5), let us consider there is an experimental or counterfactual intervention on neurons *v*_1_, …, *v*_*k*_ ∈ *V* during the set of times *I* ⊆ {0, 1, …, *T*}, such as ablation or external control. 1) For ablation of *v*_1_, …, *v*_*k*_ the connections incident as well as outgoing from them are removed. 2) During *t* ∈ *I*, for external control, all connections incident on *v*_1_, …, *v*_*k*_ are removed in the Rolled CFC-DPGM *F*_*τ*_ and other connections remain intact.

*Proof*. See Section B in S1 Appendix.

This corollary justifies the usage of causal reasoning with the edges of the CFC alone to answer the interventional queries. Answers to the questions by causally reasoning are as follows:

1. When neuron *A* is ablated, one just deletes all the edges incident and originating from neuron *A* since *A* has a fixed value after ablation and neither do other neurons influence the activity of neuron *A* nor does *A* influence the activity of any other neuron.
2. When activity of neuron *B* is externally controlled, one simply removes the edges incident on neuron *B*, because activity of neuron *B* no longer depends on its parent neurons in the CFC obtained before intervention rather the activity of neuron *B* depends on the external control. Edges originating from neuron *B* in the CFC from before the intervention should remain intact during the intervention since the functional pathways from *B* to its descendant neurons in the CFC remain intact during external control. To illustrate the properties of non-parametric causal relations (Theorem 1) and interventions (Corollary 5.1), we consider the following example.

**Fig 8:**
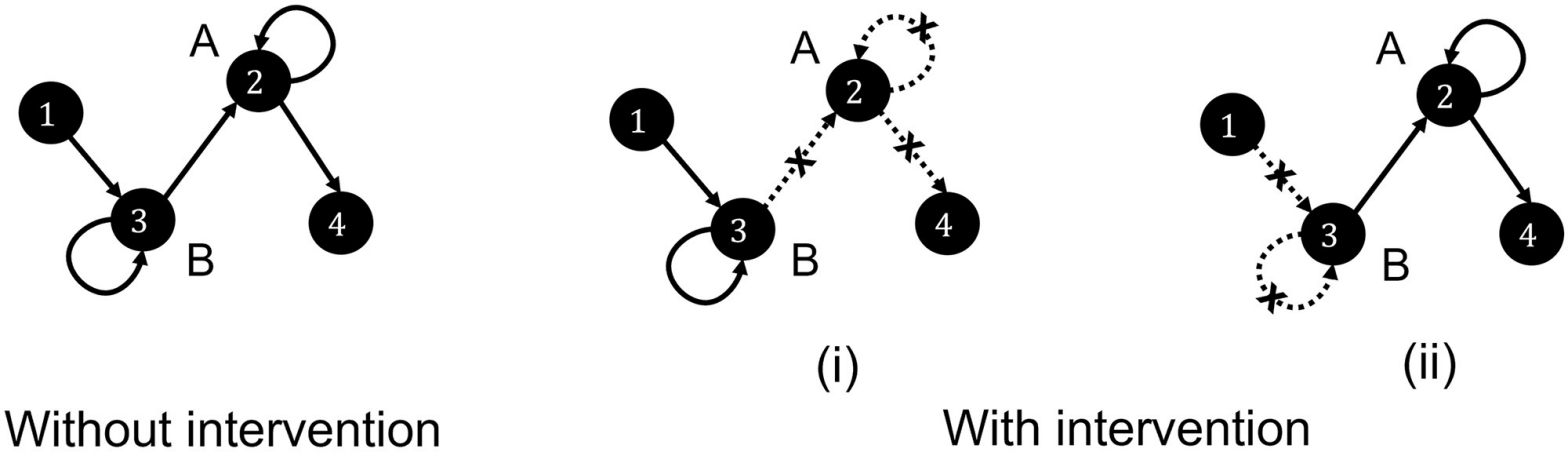
Impact of intervention. Rolled CFC-DPGM (left) for neurons 1–4 with dynamics as in Example 5.1, and consequence of intervention on neurons labelled A and B by (i) Ablation of A and (ii) External modulation of B.

**Example 5.1** Let *V* denote a network of 4 neurons, labeled {1, 2, 3, 4} with neural activity *X*_*v*_(*t*), *v* ∈ *V* related as,

$$\begin{array}{cc} X_{1}\left(t\right) & =g_{1,t}\left(\epsilon_{1}\left(t\right)\right)\\ X_{2}\left(t\right) & =g_{2,t}\left(X_{2}\left(t-1\right),X_{3}\left(t-1\right),X_{3}\left(t-2\right),\epsilon_{2}\left(t\right)\right)\\ X_{3}\left(t\right) & =g_{3,t}\left(X_{3}\left(t-1\right),X_{1}\left(t-1\right),\epsilon_{3}\left(t\right)\right)\\ X_{4}\left(t\right) & =g_{4,t}\left(X_{2}\left(t-1\right),\epsilon_{4}\left(t\right)\right) \end{array}$$

for independent random variables *ϵ*_*v*_(*t*) and measurable functions *g*_*v*,*t*_, *v* = 1, 2, 3, 4; 0 ≤ *t* ≤ 1000 msec. By Theorem 1 it follows that the graph: 2 → 2, 3 → 2, 3 → 3, 1 → 3, 2 → 4 as in Fig 8-left is the CFC among the neurons considering maximum time delay of interaction to be 1 msec or higher. Suppose one asks the question of type (i), how would the functional connectome change if neuron (A) were ablated? According to Corollary 5.1, the resulting CFC would be 1 → 3, 3 → 3 as in Fig 8-middle by removing the connections to and from neuron 2 according to Corollary 5.1. Suppose one asks the question of type (ii), how would the functional connectome change if activity of neuron (B) were to be externally controlled by optogenetics? According to Corollary 5.1, the resulting CFC would be 3 → 2, 2 → 2, 2 → 4 as in Fig 8-right by removing the parent connections of neuron 3 according to Corollary 5.1.

## Supporting information

S1 Appendix Proofs and Datasets. A. Proof of Theorem 1. B. Proof of Corollary 7.1. C. Simulation Study Details. D. Benchmark Datasets. E. Visual Coding Neuropixels Dataset.
(PDF)
