# Supplementary material for: Statistical perspective on functional and causal neural connectomics: The Time-Aware PC algorithm
Source: PLoS Comput Biol. 2022 Nov 14;18(11):e1010653. doi: 10.1371/journal.pcbi.1010653 (PMC9704761; doi:10.1371/journal.pcbi.1010653)
Supplement: S1 Appendix — A. Proof of Theorem 1. B. Proof of Corollary 7.1. C. Simulation Study Details. D. Benchmark Datasets. E. Visual Coding Neuropixels Dataset. (PDF) [file pcbi.1010653.s001.pdf]

Statistical Perspective on Functional and Causal Neural Connectomics: The Time-Aware PC Algorithm

Rahul Biswas
Department of Statistics
University of Washington
Seattle, WA, 98195
rbiswas1@uw.edu

Eli Shlizerman
Department of Applied Mathematics
Department of Electrical & Computer Engineering
University of Washington
Seattle, WA, 98195
shlizee@uw.edu

Proofs and Datasets

A Proof of Theorem 1

Let $\boldsymbol{V} = \{(v,t) : v \in V, t \in T\}$, $\boldsymbol{E} = \{(u_{v,i}, t_{v,i}) \to (v,t) : 1 \le i \le p, v \in V, t \in \{0,1,\ldots,T\}\}$, and $\boldsymbol{G} = (\boldsymbol{V}, \boldsymbol{E})$. Rewriting Eq. (5) we get,

$$\boldsymbol{X}(v,t) = g_{v,t}(\{\boldsymbol{X}(u,k) : (u,k) \in pa_{\boldsymbol{G}}((v,t))\}, \epsilon_v(t))$$

By Theorem 1.4.1 in [1], the above implies that $\boldsymbol{X}$ satisfies the DMP with respect to $\boldsymbol{G}$.

Therefore by Definition 1, the rolled CFC-DPGM, F_τ, has nodes V and edges given by $u_{v,i} \to v$, since $(u_{v,i}, t_{v,i}) \to (v,t) \in \boldsymbol{E}$, for $v \in V$ and $1 \le i \le K$. That is, $pa_{F_\tau}(v) = \{u_{v,1}, \ldots, u_{v,K}\}$.

B Proof of Corollary 7.1

For $t \in I$, i.e. during the experimental/counterfactual intervention, such as controlling the activity of neuron or neuron ablation, the activity $X_{v_1}(t), \ldots, X_{v_k}(t)$ would no longer be a function of the activities of the neurons at preceding time points, while the activity of neurons which are not intervened, would still be a function of activity at preceding time points. That is, $X_v(t) = g_{v,t}(X_{pa_{F_\tau}(v)}(t-), \epsilon_v(t))$, for $v \notin \{v_1, \ldots, v_k\}$ and $X_v(t) = g_{v_i,t}(\epsilon_{v_i}(t))$ for $v \in \{v_1, \ldots, v_k\}$ for $t \in I$, where $g_{v_i,t}(\epsilon_{v_i}(t))$ represents the distribution of neural activity due to the experimental intervention. For example, $g_{v_i,t}$ is identically 0 for neuron ablation, and can be an oscillating function with a high amplitude and random noise $\epsilon_{v_i}(t)$ for stimulation through external control. The dynamics can be written as

$$X_v(t) = g_{v,t}(X_{pa_{F'_\tau}(v)}(t-), \epsilon_v(t)) \quad (1)$$

for $v \in V, t \in I$, where $pa_{F'_\tau}(v) = pa_{F_\tau}(v)$ for $v \notin \{v_1, \ldots, v_k\}$ and $pa_{F'_\tau}(v_1) = \ldots = pa_{F'_\tau}(v_k) = \Phi$, where Φ denotes the null set. In other words, F'_τ has all connections same as F_τ except that all connections directing to the intervened neurons $v_1, \ldots, v_k$ are removed. It follows

from Eq. (1) and Theorem 1 that F'_τ is the causal functional connectivity between the neurons in V during time I when $v_1, \ldots, v_k$ are subject to experimental intervention.

For ablations,the edges originating from $v_1, \ldots, v_k$, can be removed since the activity of these neurons would be fixed (at zero) and would be trivial variables that can be excluded from the argument of the function $g_{v,t}$ in Eq. (1).

C Simulation Study Details

We study the following simulation paradigms.

1. Linear Gaussian Time Series (Figure 3a left-column). Let $N(0, \eta)$ denote a Normal random variable with mean 0 and standard deviation η. We define $X_v(t)$ as a linear Gaussian time series for $v = 1, \ldots, 4$ whose true CFC has the edges $1 \to 3, 2 \to 3, 3 \to 4$. Let $X_v(0) = N(0, \eta)$ for $v = 1, \ldots, 4$, and for $t = 1, 2, \ldots, 1000$,

$$X_1(t) = 1 + N(0, \eta), \qquad X_2(t) = -1 + N(0, \eta),$$
$$X_3(t+1) = 2X_1(t) + X_2(t) + N(0, \eta), \qquad X_4(t+1) = 2X_3(t) + N(0, \eta).$$

We obtain 25 simulations of the entire time series each for different noise levels $\eta \in \{0.1, 0.5, 1, 1.5, 2, 2.5, 3, 3.5\}$.

2. Non-linear Non-Gaussian Time Series (Figure 3a middle-column). Let $U(0, \eta)$ denote a *Uniformly* distributed random variable on the interval $(0, \eta)$. We define $X_v(t)$ as a non-linear non-Gaussian time series for $v = 1, \ldots, 4$ whose true CFC has the edges $1 \to 3, 2 \to 3, 3 \to 4$. Let $X_v(0) = U(0, \eta)$ for $v = 1, \ldots, 4$ and for $t = 1, 2, \ldots, 1000$,

$$X_1(t) = U(0, \eta), \qquad X_2(t) = U(0, \eta),$$
$$X_3(t+1) = 4\sin(X_1(t)) + 3\cos(X_2(t)) + U(0, \eta), \qquad X_4(t+1) = 2\sin(X_3(t)) + U(0, \eta).$$

We obtain 25 simulations of the entire time series each for different noise levels $\eta \in \{0.1, 0.5, 1, 1.5, 2, 2.5, 3, 3.5\}$.

3. Continuous Time Recurrent Neural Network (CTRNN) (Figure 3a right-column). We simulate neural dynamics by Continuous Time Recurrent Neural Networks, Eq. (2). $u_j(t)$ is the instantaneous firing rate at time t for a post-synaptic neuron j, w_{ij} is the linear coefficient to pre-synaptic neuron i's input on the post-synaptic neuron j, $I_j(t)$ is the input current on neuron j at time t, τ_j is the time constant of the post-synaptic neuron j, with i, j being indices for neurons with m being the total number of neurons. Such a model is typically used to simulate neurons as firing rate units,

$$\tau_j \frac{du_j(t)}{dt} = -u_j(t) + \sum_{i=1}^{m} w_{ij}\sigma(u_i(t)) + I_j(t), j = 1, \ldots, m. \tag{2}$$

We consider a motif consisting of 4 neurons with $w_{13} = w_{23} = w_{34} = 10$ and $w_{ij} = 0$ otherwise. We also note that in Eq. 2, activity of each neuron $u_j(t)$ depends on its own past. Therefore, the true CFC has the edges $1 \to 3, 2 \to 3, 3 \to 4, 1 \to 1, 2 \to 2, 3 \to 3, 4 \to 4$. The time constant τ_i is set to 10 msecs for each neuron i. We consider $I_i(t)$ to be distributed as independent Gaussian process with the mean of 1 and the standard deviation of η. The signals are sampled at a time gap of $e \approx 2.72$ msecs for a total duration of 1000 msecs. We obtain 25 simulations of the entire time series each for different noise levels $\eta \in \{0.1, 0.5, 1, 1.5, 2, 2.5, 3, 3.5\}$.

The GC graph is computed using the *Nitime* Python library, which fits an MVAR model followed by using the *GrangerAnalyzer* to compute the Granger Causality [2]. The PC algorithm, which requires several samples of a scalar-valued random variable Y_v (measured activity) for neurons $v \in V$, is used to compute DPGM. We define Y_v as a windowed average of recordings over a duration of 50 msec: $Y_v = X_v, v \in V$, and averaging over different 50 msec windows with a gap of 50 msec between consecutive windows yields different Y_v samples. This choice of Y_v performs better than considering Y_v to be neural recordings at time t: $Y_v = X_v(t), v \in V$, with different t giving different samples of Y_v in previous work [3]. The TPC algorithm computes the rolled CFC-DPGM directly from the signals and, we use a maximum time-delay of interaction of 1 msec.

The choice of thresholds tunes the decision whether a connection exists in the CFC. For DPGM and TPC, increasing the significance level α for conditional independence tests increases the rate of detecting edges, but also increasing the rate of detecting false positives. We consider $\alpha = 0.01, 0.05, 0.1$ for DPGM and TPC. For GC, a likelihood ratio statistic L_{uv} is obtained for testing $A_{uv}(k) = 0$ for $k = 1, \ldots, K$. An edge $u \rightarrow v$ is outputted if L_{uv} has a value greater than a threshold. We use a percentile-based threshold, and output an edge $u \rightarrow v$ if L_{uv} is greater than $100(1 - \alpha)$ percentile of L_{ij}'s over all pairs of neurons (i, j) in the graph [4]. We consider $\alpha = 0.01, 0.05, 0.1$ which corresponds to percentile thresholds of $99\%, 95\%, 90\%$. For the bootstrap procedure in TPC, we consider 50 bootstrap iterations with bootstrap window length of 50 msec and bootstrap stability cutoff $\gamma = 25\%$.

D Benchmark Datasets

We use the following benchmark datasets from *Causeme* [5, 6].

1. River Runoff. This is a real dataset that consists of time series of river runoff at different stations. The time series have a daily time resolution and only include summer months (June-August). The physical time delay of interaction (as inferred from the river velocity) are roughly below one day, hence the dataset has contemporaneous time interactions. This dataset has 12 variables and 4600 time recordings for each variable.

2. Logistic Map. This is a synthetic dataset generated from logistic map with maximum time delay of 3 and a low dynamical noise and moderate strength of coupling between the variables. This dataset has 5 variables and 300 time recordings per variable.

The PC algorithm is used to compute the DPGM from the scalar-valued random variables Y_v, defined by the average of recordings of a time window of length Δt, and averaging over alternate time windows of length Δt and a gap of Δt between consecutive windows results in samples of Y_v. Considering river runoff has large number of 4600 time recordings while logistic map has only 300 recordings, we used $\Delta t = 50$ and 3 for river runoff and logistic map data respectively. The PC algorithm was implemented with p-value of 0.1 for kernel-based non-linear conditional independence tests. In river-runoff and logistic map data, the TPC algorithm was implemented with maximum time-delay of interaction to be 1 and 3 recordings respectively, as per specification in the datasets, and a significance level $\alpha = 0.1$ for kernel-based conditional independence tests. For the bootstrap procedure in TPC, 50 bootstrap iterations with bootstrap window length of 50 recordings and bootstrap stability threshold $\gamma = 0.01, 0.15$ for river-runoff and logistic map datasets respectively.

E Visual Coding Neuropixels Dataset

For the purpose of application and comparison of the results of the methods discussed in this paper, we restrict our analysis to a 116 days old male mouse (Session ID 791319847) with 555 neurons whose spike trains are recorded simultaneously by six Neuropixel probes. The spike trains during the entire experiment were recorded at a frequency of 1 KHz. We analyze the spike trains for four stimuli categories:

1. Natural scenes, consisting of 118 natural scenes selected from three databases (Berkeley Segmentation Dataset, van Hateren Natural Image Dataset and McGill Calibrated Colour Image Database), with each scene presented briefly for 250ms and then replaced with the next scene image. Each scene is repeated 50 times in random order with intermittent blank intervals.
2. Static gratings consisting of full-field sinusoidal gratings with 6 orientations (the angle of the grating), 5 spatial frequencies (the width of the grating), and 4 phases (the position of the grating) resulting in 120 stimulus conditions. Each grating is presented briefly (250 ms) before being replaced with a different orientation, spatial frequency and phase condition and each condition is repeated 50 times, in random order with intermittent blank intervals.
3. Gabor patches with 3 orientations where the patch center is lying at one of the points in a 9×9 visual field. Each Gabor patch is being presented for 250ms and then replaced by a different patch, and each condition is repeated 50 times in random order with intermittent blank intervals.
4. Full-field flashes, lasting for 250 ms followed by a blank interval of 1.75 s, and then the next flash, totaling 150 repetitions.

This variety of stimuli is ranging from relatively *natural stimuli* invoking mice's natural habitats (natural scenes) to *artificial stimuli* (static gratings, Gabors and flashes). Among the artificial stimuli, static gratings incorporate sinusoidal patches, while full-field flashes incorporate sharp changes in luminosity in the whole visual field in short period of time, and Gabor patches incorporate sinusoidal patches with declining luminosity with distance from the center of the patch. With this choice of four stimuli we investigate how the variety of stimuli possibly invokes distinct patterns of neuronal interactions and connectivity. We exclude dynamic stimuli like natural movies, and drifting gratings, from this analysis because their results would require more nuanced study and interpretation, which we defer for future analysis.

Preprocessing We convert the spike trains recorded at 1 KHz to bin size of 10 ms by aggregating and then separating by start and end times of each stimuli presentation and obtain the Peri-Stimulus Time Histograms (PSTH) with bin-size 10 ms. We smooth the PSTHs by a Gaussian smoothing kernel of bandwidth 16ms which provides a smoothed version of the PSTH for each neuron and each stimulus presentation. Some examples of the smoothed PSTH are displayed in Figure 6. We use the smoothed PSTHs for neurons over each stimuli type as input for inference of the FC between the neurons for each stimuli presentation. For each stimulus presentation, we first selected the set of neurons that were active in at least 25% of the bins in the PSTH, and then collected the set of unique neurons over all stimuli, which resulted in 54, 43, 19 and 36 active neurons for natural scenes, static gratings, Gabor patches and flashes respectively, and 68 unique active neurons overall. We considered separated the entire duration of stimulus presentation to yield 58 trials of natural scenes, 60 trials of static gratings, 58 trials of Gabor patches, and, 3 trials of flashes, where each trial is of duration 7.5 s.

We compare TPC with two popular methods for inferring the FC from neural signals: Granger Causality (GC) and Sparse Partial correlation via Graphical Lasso penalized Maximum Likelihood

Estimation (Sparse Partial Corr). The TPC algorithm was implemented with maximum time-delay of interaction 10 ms, significance level $\alpha = 0.3$, 50 bootstrap iterations, 250 ms bootstrap window width, and stability threshold $\gamma = 0.01$. For GC, we consider VAR model of order 1, and GC likelihood ratio statistic of greater than 90 percentile as indicating edges [4]. For Sparse Partial Corr, the optimal penalization was determined by 5-fold cross-validation. A summary of the results is provided in Figure 6.

References

[1] Judea Pearl. *Causality*. Cambridge university press, 2009.

[2] Ariel Rokem, M Trumpis, and F Perez. Nitime: time-series analysis for neuroimaging data. In *Proceedings of the 8th Python in Science Conference*, pages 68–75, 2009.

[3] Rahul Biswas and Eli Shlizerman. Statistical perspective on functional and causal neural connectomics: A comparative study. *Frontiers in Systems Neuroscience*, 16, 2022.

[4] Christoph Schmidt, Britta Pester, Nicole Schmid-Hertel, Herbert Witte, Axel Wismüller, and Lutz Leistritz. A multivariate granger causality concept towards full brain functional connectivity. *PloS one*, 11(4):e0153105, 2016.

[5] Jakob Runge, Sebastian Bathiany, Erik Bollt, Gustau Camps-Valls, Dim Coumou, Ethan Deyle, Clark Glymour, Marlene Kretschmer, Miguel D Mahecha, Jordi Muñoz-Marí, et al. Inferring causation from time series in earth system sciences. *Nature communications*, 10(1):1–13, 2019.

[6] Bart Bussmann, Jannes Nys, and Steven Latré. Neural additive vector autoregression models for causal discovery in time series. In *International Conference on Discovery Science*, pages 446–460. Springer, 2021.
